# 20-Hydroxyecdysone-Loaded Niosomes as a Novel Nano-Therapeutic Platform for Psoriasis: In Vitro Characterization and *Danio rerio* Toxicity Studies

**DOI:** 10.3390/pharmaceutics18091096

**Published:** 2026-08-31

**Authors:** Ludwika Piwowarczyk, Jagoda Szkudlarek, Dariusz T. Mlynarczyk, Szymon Tomczak, Violetta Krajka-Kuźniak, Aleksandra Majchrzak-Celińska, Robert Kleszcz, Ewelina Musielak, Mateusz de Mezer, Emilia Cicha, Gabriela Anglart, Anna Jelińska

**Affiliations:** 1 Poznan University of Medical Sciences, Department of Pharmaceutical Chemistry, Rokietnicka 3, 60-806 Poznan, Poland; szymon.tomczak@ump.edu.pl (S.T.); gabriela.anglart@gmail.com (G.A.); ajelinsk@ump.edu.pl (A.J.); 2 Poznan University of Medical Sciences, Doctoral School, Bukowska 70, 60-812 Poznan, Poland; jszkudlarek@ump.edu.pl (J.S.); 3 Poznan University of Medical Sciences, Department of Chemical Technology of Drugs, Rokietnicka 3, 60-803 Poznan, Poland; mlynarczykd@ump.edu.pl; 4 Poznan University of Medical Sciences, Department of Pharmaceutical Biochemistry, Rokietnicka 3, 60-806 Poznan, Poland; majchrzakcelinska@ump.edu.pl (A.M.-C.); kleszcz@ump.edu.pl (R.K.); emusielak@ump.edu.pl (E.M.); vkrajka@ump.edu.pl (V.K.-K.); 5 Poznan University of Medical Sciences, Department of Immunobiology, Rokietnicka 8, 60-806 Poznan, Poland; mdemezer@ump.edu.pl; 6 Poznan University of Medical Sciences, Animal Facility, 60-806 Poznan, Poland; emiliacicha@ump.edu.pl

**Keywords:** 20-hydroxyecdysone, niosomes, drug delivery systems, keratinocytes, psoriasis, *Danio rerio*

## Abstract

**Background/Objectives:** Psoriasis is an incurable chronic immune-mediated inflammatory skin disorder for which effective topical treatment remains essential. Given the reported anti-inflammatory and dermatological potential of 20-hydroxyecdysone (20HE), this study evaluated its biological activity in normal and psoriatic keratinocytes and developed stable 20HE-loaded niosomes intended for potential future dermal delivery. **Methods:** The biological activity of 20HE was assessed in human epidermal keratinocytes (HEKs) and psoriasis-patient-derived keratinocytes (PHEKs) after 48 h of treatment by quantifying selected cytokines. Niosomes were prepared by thin-film hydration, rehydrated with water or citrate buffer, and sonicated. The formulations were characterized for particle size, polydispersity index, zeta potential, pH, encapsulation efficiency (EE), and 21-day storage stability. Potential in vivo toxicity was assessed in *Danio rerio* embryos by monitoring mortality, sublethal developmental changes, and locomotor behavior after exposure to nanoformulations for up to 96 h post-fertilization. **Results:** 20HE showed selective anti-inflammatory activity in PHEK cells, reducing IL-4, IL-17A, and IL-22 levels by 20–34% under elevated inflammatory conditions, without affecting healthy HEK cells. The formulations had particle sizes below 300 nm and acceptable polydispersity and zeta potential values, and they remained stable during storage. In the *Danio rerio* embryo model, the 1A/water caused sublethal developmental abnormalities at 96 hpf, whereas 1C/water (with 20HE) was associated only with mild pericardial edema after prolonged exposure. No significant behavioral alterations were observed across the tested concentration range, and the Lowest Observed Effect Concentration for 20HE was 20 μM. **Conclusions:** Free 20HE reduced the levels of IL-4, IL-17A, and IL-22 in PHEK cells, indicating its modulatory effect on cytokine production under the investigated inflammatory conditions. The developed 20HE-loaded niosomes showed favorable physicochemical properties and short-term stability, supporting their potential as candidate nanocarrier systems for future dermal application studies. The findings of the present study provide a basis for further investigation, particularly studies on drug release from the niosomal formulation and skin permeation from the final pharmaceutical dosage form.

## 1. Introduction

Psoriasis is a chronic immune-mediated inflammatory skin disease characterized by excessive keratinocyte proliferation, inflammatory cell infiltration, and alterations in programmed cell death pathways [1]. Its prevalence varies considerably across geographical regions. Among adults, physician-diagnosed lifetime prevalence has been estimated to range from 0.14% (95% uncertainty interval [UI]: 0.05–0.40%) in East Asia to 1.99% (95% UI: 0.64–6.60%) in Australasia. Relatively high prevalence estimates have also been reported in Western Europe (1.92%, 95% UI: 1.07–3.46%), Central Europe (1.83%, 95% UI: 0.62–5.32%), North America (1.50%, 95% UI: 0.63–3.60%), and high-income Southern Latin America (1.10%, 95% UI: 0.36–2.96%) [2]. In psoriasis, external stimuli activate skin dendritic cells, which subsequently release interleukin 23 (IL-23). This release bridges innate and adaptive immunity, triggering the differentiation of T helper 17 (Th17) cells and increasing interleukin 17 (IL-17) production, which in turn stimulates excessive keratinocyte production. This autoimmune cycle in the pathogenesis of psoriasis is modulated by G protein-coupled receptor (GPCR) signaling pathways [3].

The condition affects both the skin and nails, with the most common signs and symptoms being skin scaling (92%), excessive itching (72%), erythema and redness (69%), fatigue and lethargy (27%), inflammation (23%), burning sensations (20%), and bleeding (20%) [4]. Patients with psoriasis endure chronic discomfort, and the condition remains incurable. Consequently, the development of effective and safe treatments for psoriasis is essential [5]. For mild cases, treatment typically involves lifestyle changes and topical therapies, including topical corticosteroids, emollients, vitamin D analogs, and calcineurin inhibitors. In cases of more severe psoriasis, systemic treatment may be necessary, with options including oral medications, such as methotrexate, cyclosporine, and acitretin, or biologic therapies [6,7]. Skin disorders, such as psoriasis, inflammation, infections, and dermatitis, often require topical therapy that targets lesions locally to maximize therapeutic efficacy while minimizing systemic absorption and adverse effects [8].

Among compounds of potential dermatological interest, ecdysteroids have attracted increasing attention. These compounds are best known as arthropod steroid hormones involved in molting, development, and reproduction. However, they are also synthesized by plants as a defense mechanism against phytophagous insects, often at levels higher than in insects (4–5%) [9]. One of the best-studied representatives is 20HE (ecdysterone, β-ecdysone, polypodine A), a polyhydroxylated steroid naturally present in small but significant quantities in invertebrates, where it serves hormonal functions, and in certain plant species, where it is thought to help deter invertebrate predators [10]. For instance, 20HE content in the leaves of *Serratula coronata* and the roots of *Leuzea carthamoides* reaches 1.5% of the plant’s dry weight [11]. Interestingly, Kroma et al. demonstrated that creams enriched with *S. coronata* extract exerted favorable effects on psoriatic skin lesions [12].

**Figure 1:**
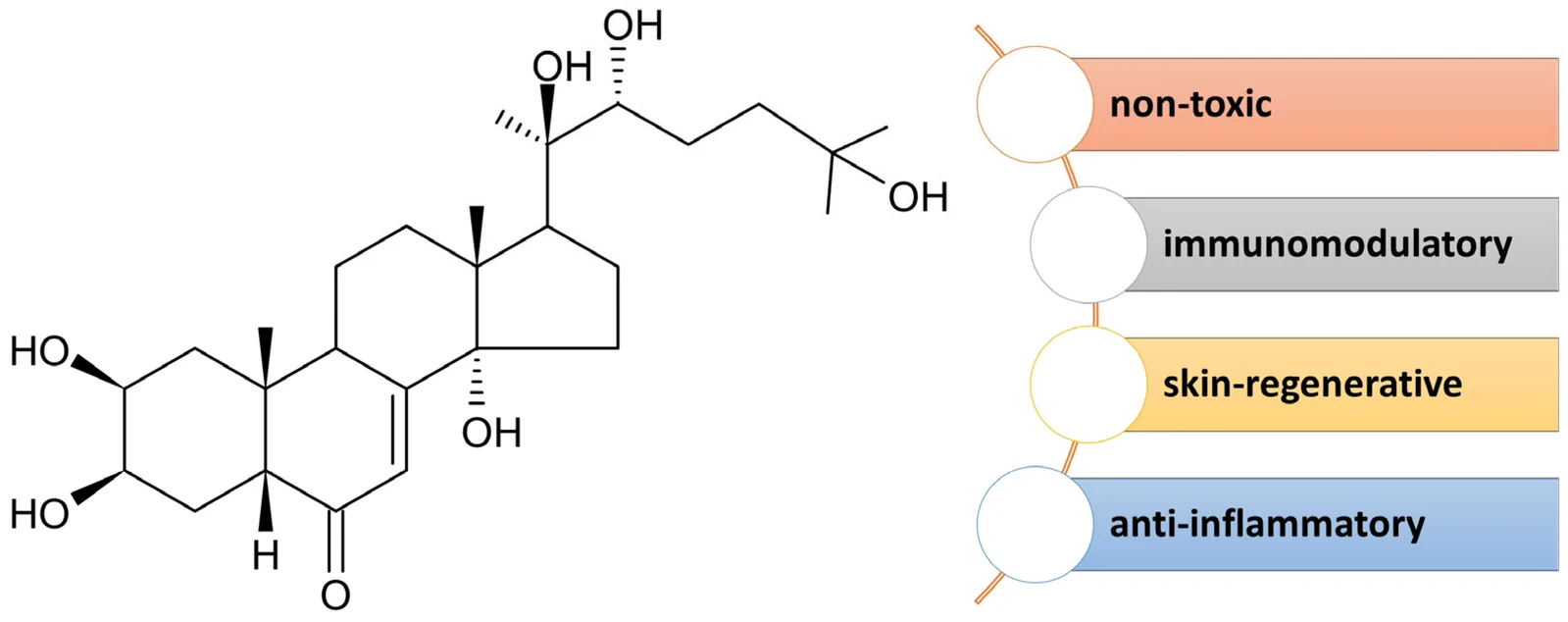
Chemical structure of 20HE and selected properties that support its potential use in psoriasis therapy.

Given its promising effects, there is increasing interest in the pharmaceutical and medical applications of 20HE. Large-scale synthesis methods for pharmaceutical-grade 20HE have been established, and its safety has been verified in regulatory preclinical studies, showing no toxic effects [10]. As noted by Lafont et al., this broad, commercial availability has facilitated the use of 20HE in dietary supplements for humans (bodybuilders, athletes) and animals (horses and dogs), as well as in cosmetic formulations designed to enhance skin quality and regeneration [13]. Moreover, 20HE is regarded as a low-toxicity compound in mammals, with an LD_50_ (in mice) of >9 g/kg when administered orally, and 6.4 g/kg after intraperitoneal injection [14]. The effects of 20HE on mammals are pleiotropic and include immunomodulatory activity. It has also been reported to exert beneficial effects on various organs, including promoting wound healing in the skin [9,15]. In skeletal muscle cells, phytoecdysteroids like 20HE stimulate protein synthesis. Gorelick-Feldman et al. demonstrated that in the C(2)C(12) mouse skeletal muscle cell line, 20HE triggered a swift rise in intracellular calcium levels, leading to prolonged Akt activation and enhanced protein synthesis. This response was inhibited by a GPCR antagonist, a phospholipase C (PLC) inhibitor, and a phosphoinositide 3-kinase (PI3K) inhibitor [16]. Further research by Ehrhardt et al. explored 20HE as a treatment for skin conditions in postmenopausal women using ovariectomized rats. They found that 20HE treatment increased the thickness of the epidermal and dermal layers compared to control and β-estradiol-treated rats, while subcutaneous fat thickness was intermediate. Muscle thickness was also greater in both 20HE and β-estradiol-treated rats [15]. Finally, Park et al. explored the effects of 20HE on UVB-irradiated HaCaT cells, a key model in dermatological research. They found that it inhibited 11β-hydroxysteroid dehydrogenase type 1 expression and enhanced glucocorticoid receptor activity [9]. Collectively, these findings indicate that 20HE is a promising candidate for dermatological and anti-inflammatory applications. Figure 1 illustrates the chemical structure of 20HE and highlights selected properties that support its potential use in psoriasis therapy.

However, effective topical therapy requires overcoming the skin’s barrier properties. The skin consists of three main layers—the epidermis, dermis, and subcutaneous tissue—which together perform protective, secretory, excretory, and absorptive functions. The outer epidermal layer, primarily composed of keratinocytes, forms the stratum corneum, the principal barrier for the penetration of substances. Nanocarrier-based topical systems enhance the delivery and bioavailability of active compounds, simultaneously improving their stability and protecting them from degradation. Owing to their small size, deformability, surface charge properties, and ability to alter stratum corneum structure, these carriers facilitate deeper skin penetration and improved therapeutic performance [17].

Vesicular drug delivery systems (DDSs) have been widely developed as promising alternatives to conventional skin therapies because they enhance dermal drug delivery, improve drug accumulation at the target site, and reduce skin irritation, drug degradation, and systemic toxicity [18]. Vesicular nanocarriers, including liposomes, transfersomes, elastosomes, and niosomes, offer high biocompatibility and can incorporate hydrophilic and lipophilic compounds. Their biomimetic bilayer structure promotes interaction and fusion with the skin. However, certain systems exhibit limited physicochemical stability, and their topical performance strongly depends on formulation composition and preparation technique [17]. To overcome the limitations (such as limited stability and premature drug leakage) of the most well-known vesicular nanosystems, e.g., liposomes, structural modifications have been introduced, resulting in the development of alternative vesicular carriers, including niosomes.

**Figure 2:**
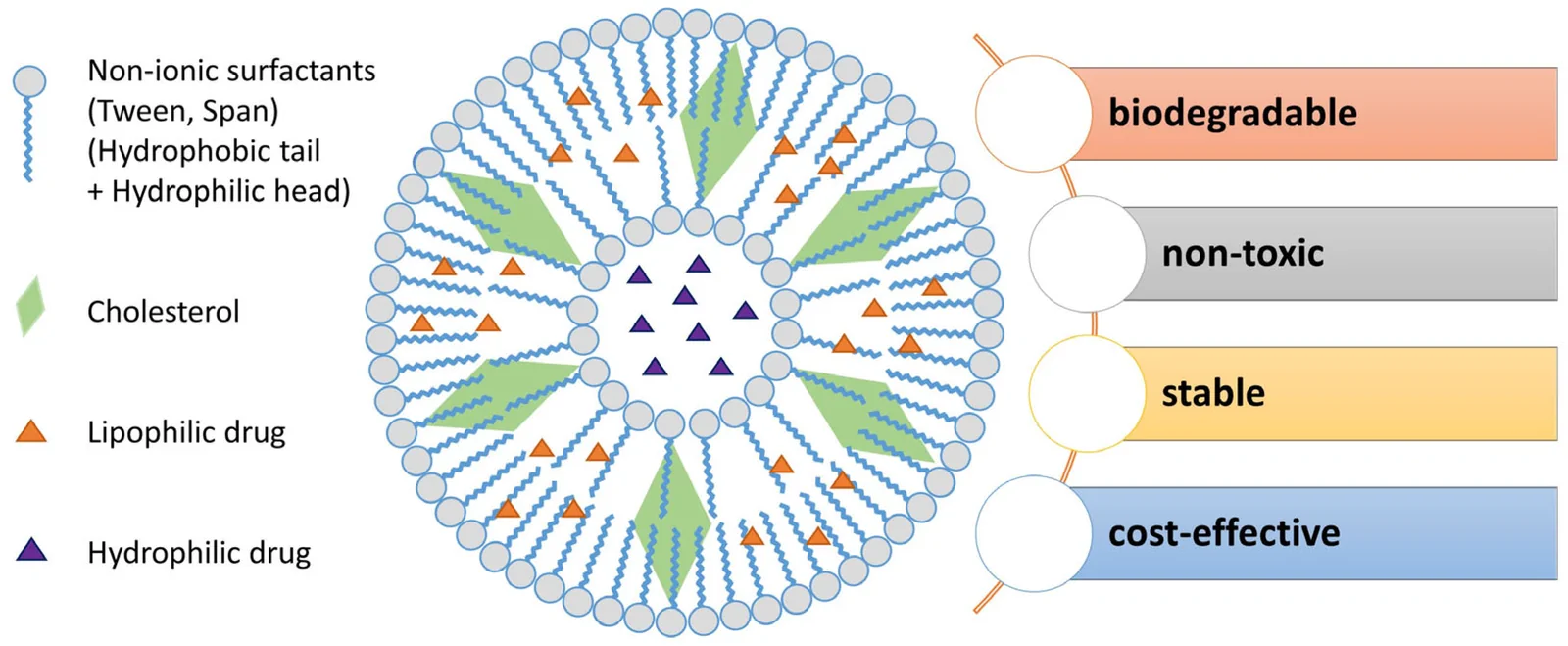
Schematic representation of niosome structure, its principal components, and selected advantages relevant to drug delivery.

Niosomes are nanoscale lamellar vesicles composed of lipid, mostly cholesterol (CHOL) and non-ionic surfactants, such as polysorbates (Tweens), sorbitan esters (Spans), and others [19]. First developed and patented by L’Oréal in 1975 [20], niosomes are biodegradable, non-toxic, more stable, and cost-effective alternatives to liposomes, capable of encapsulating various drugs [21]. Based on lamellarity and size, niosomes are classified into small unilamellar vesicles (single bilayer, 10–100 nm), large unilamellar vesicles (single bilayer, >100 nm), and multilamellar vesicles (multiple bilayers, >50 nm) [19]. Figure 2 illustrates the general structure of niosomes, their principal components, and selected properties that make them promising drug-delivery carriers.

Niosomes have several features that make them particularly attractive for topical delivery. Niosomes, like liposomes, alter the membrane properties of the stratum corneum and can merge with the upper layer, thereby facilitating enhanced drug permeation through the skin [22]. Topical application of niosomes extends the residence time of drugs in the stratum corneum and epidermis while minimizing systemic absorption. Niosomes affect the stratum corneum by reducing water loss and modulating skin lipid organization, enhancing smoothness. Additionally, aqueous-based vesicular suspensions, such as niosomes, provide better patient compliance than oil-based systems [23]. A stronger affinity between CHOL and skin phospholipids drives their enhanced pharmacokinetic and pharmacodynamic profiles. This interaction improves the absorption of the active ingredient but also prolongs its activity, as the complex gradually releases the active substance. Overall, they offer several benefits over liposomes, including greater chemical stability, inherent skin-penetrating properties, and reduced production costs [24].

**Figure 3:**
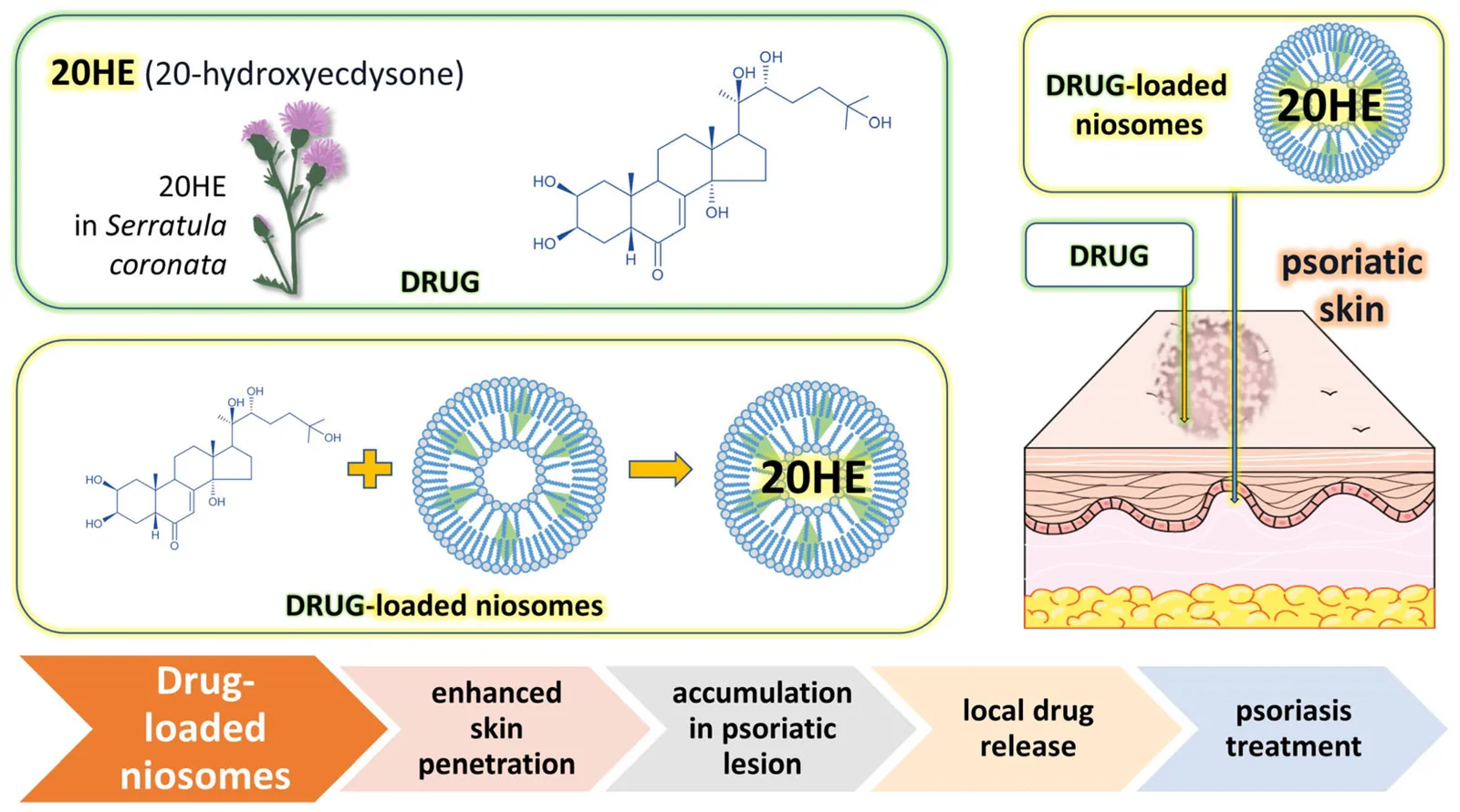
Proposed research concept of using 20HE-loaded niosomes to improve topical delivery to psoriatic skin.

The present study aimed to assess the biological activity of 20HE in the context of psoriasis by analyzing selected inflammatory cytokines in PHEKs and normal HEK. In addition, the study focused on the development and optimization of 20HE-loaded niosomal formulations for topical psoriasis treatment, including cytotoxicity assessment, evaluation of hydration media, EE, physicochemical properties, storage stability, and the suitability of selected formulations for in vivo testing in the *Danio rerio* model. Figure 3 outlines the proposed research concept of using 20HE-loaded niosomes to improve topical delivery to psoriatic skin.

Although niosomal systems have previously been investigated as topical carriers for several antipsoriatic agents, their application for the delivery of 20HE remains largely unexplored. To the best of our knowledge, this study represents the first investigation of 20HE-loaded niosomes as a potential topical formulation for psoriasis, integrating physicochemical properties analysis with assessment of biological activity in a psoriatic keratinocyte model and preliminary in vivo safety evaluation.

## 2. Materials and Methods

### 2.1. Assessment of 20HE Biological Activity Based on Cytokine Levels

HEK and PHEK cells were maintained in KGM-Gold™ Keratinocyte Growth Medium BulletKit™ (Lonza, Walkersville, MD, USA) supplemented with 10% FBS. Cells were treated with 20HE at a concentration of 20 µM (selected based on the MTS viability assay as the highest non-cytotoxic concentration) or hydrocortisone (positive anti-inflammatory control) for 48 h before cytokine analysis.

**Figure 4:**
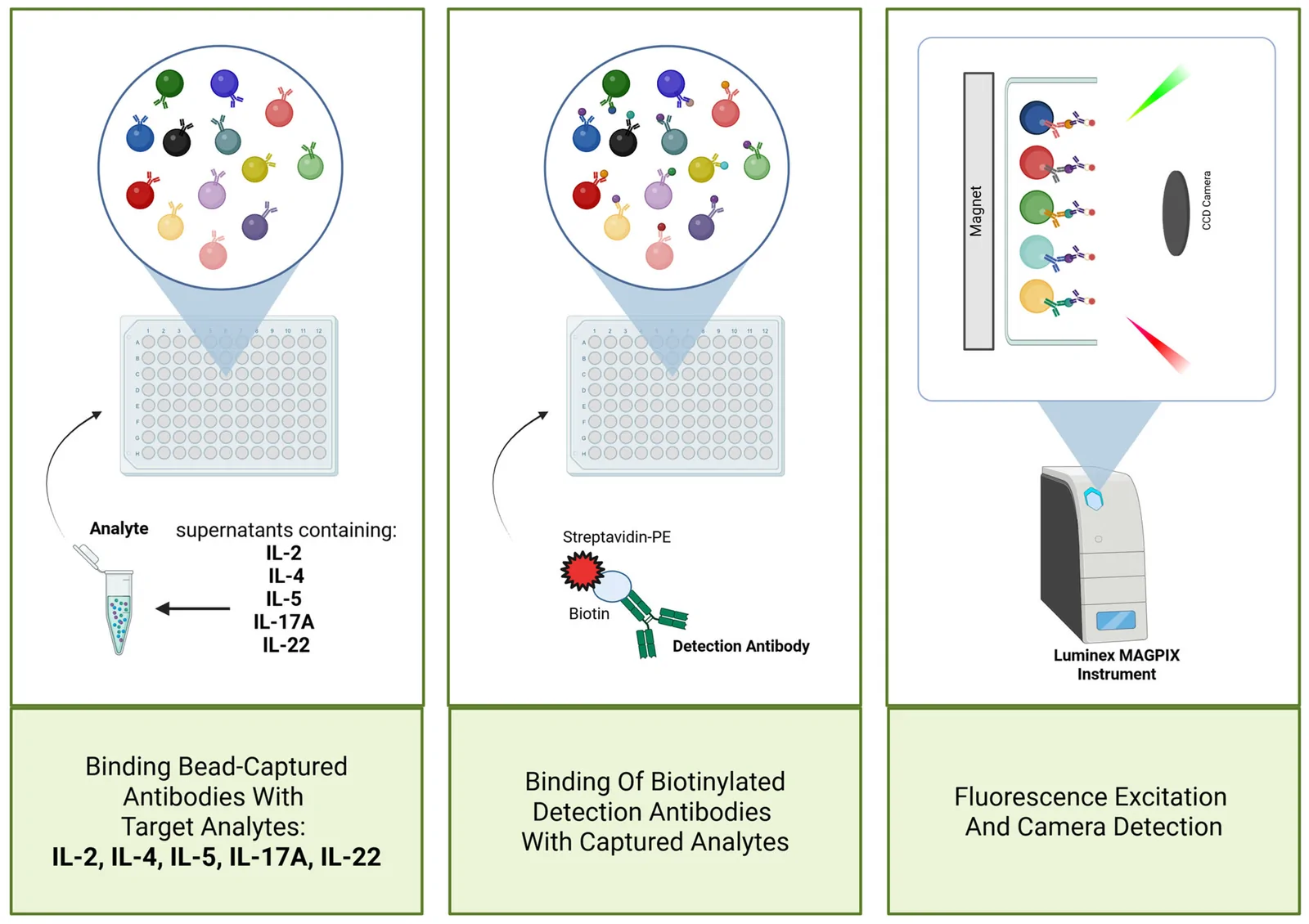
Luminex MAGPIX^®^ multiplex immunoassay analysis. Created in BioRender. Krajka-Kuźniak V. (2026) https://BioRender.com/62861dg.

A magnetic bead-based immunoassay was performed on the Luminex MAGPIX^®^ multiplex immunoassay system. We performed quantitative analysis of IL-2, IL-4, IL-5, IL-17A, and IL-22 in the supernatants according to the manufacturer’s specifications, the lower limits of detection for the analyzed cytokines were as follows: IL-2—7.1 pg/mL, IL-4—12.0 pg/mL, IL-5—11.0 pg/mL, IL-17A—2.22 pg/mL, and IL-22—22.0 pg/mL. These cytokines were selected due to their reported involvement in psoriasis-associated inflammatory signaling [25]. The magnetic beads were conjugated with specific antibodies for the protein under analysis. Using MILLIPLEX^®^ MAP Streptavidin/Phycoerythrin (SAPE) bound with MILLIPLEX^®^ MAP Detection Antibodies, a fluorescent signal was reported. The principle of the Luminex MAGPIX^®^ multiplex immunoassay analysis is shown in Figure 4. The statistical analysis conducted to determine significant differences relative to the control treatment was performed using a Dunnett test using GraphPad Instat version 3.10 (GraphPad Software, San Diego, CA, USA). Values of *p* < 0.05 were considered significant.

### 2.2. Niosome Preparation

20HE, (2*S*,3*R*,5*R*,9*R*,10*R*,13*R*,14*S*,17*S*)-2,3,14-trihydroxy-10,13-dimethyl-17-[(2*R*,3*R*)-2,3,6-trihydroxy-6-methylheptan-2-yl]-2,3,4,5,9,11,12,15,16,17-decahydro-1*H*-cyclopenta[a]phenanthren-6-one, was obtained from Carl Roth, Karlsruhe, Germany)/Beta-Ecdysterone (20-Hydroxyecdysone), European Pharmacopeia (EP) Reference Standard (Supelco, Bellefonte, PA, USA). CHOL (CHOL tested according to Ph. Eur., Sigma Aldrich, Amsterdam, The Netherlands). Tween 20 (Tween 20, BP, Ph. Eur., USP/INF grade, Glantham Life Sciences, Corsham, UK). Span 80 (Span 80 nonionic surfactant, Sigma Aldrich, USA). Anhydrous ethanol was obtained from Sigma-Aldrich (St. Louis, MO, USA) to prepare stock solutions for niosome preparation.

**Figure 5:**
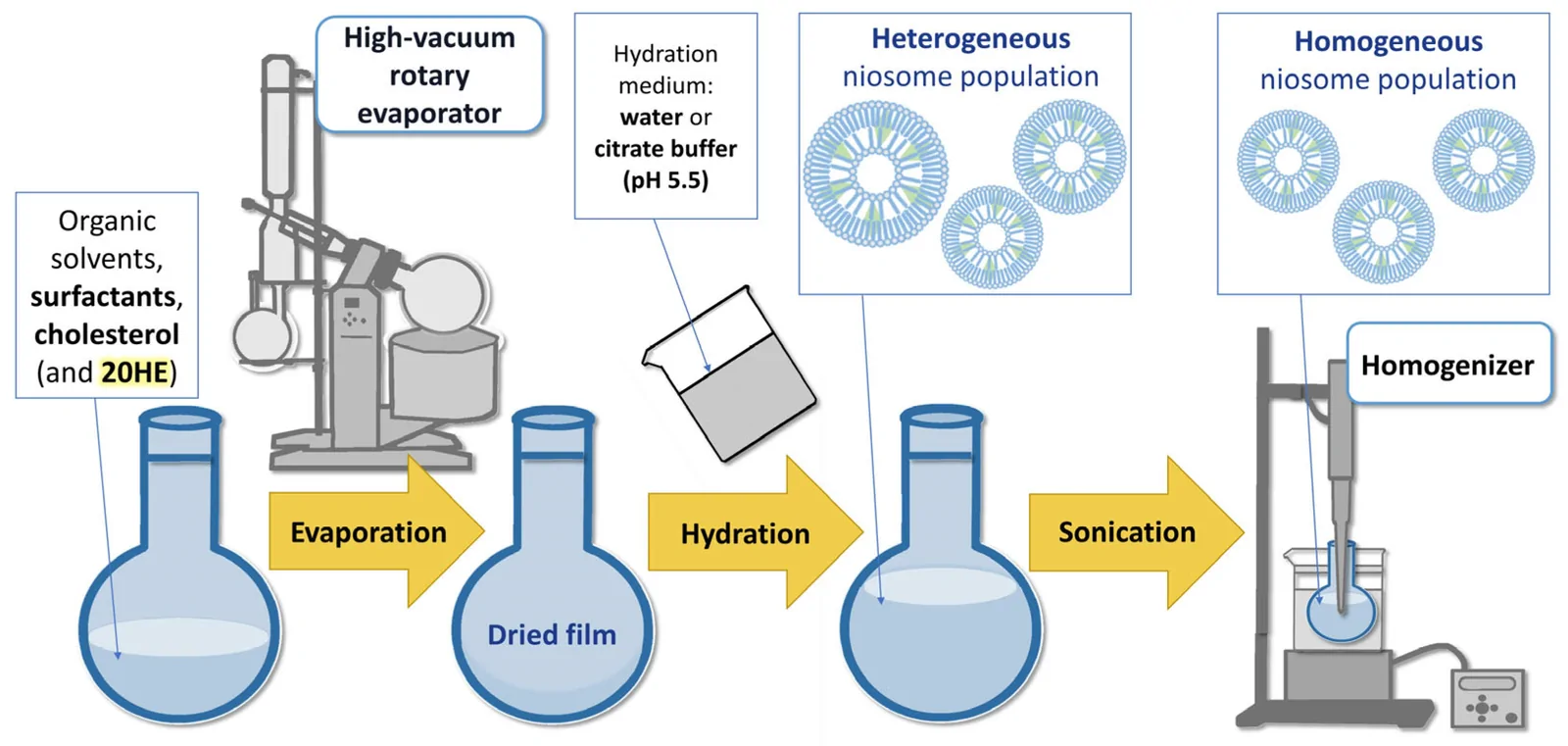
Schematic illustration of the niosome preparation protocol using thin-film hydration followed by sonication.

The procedure was based on the thin-film hydration method with sonication, with some modifications [26]. The ingredients were dissolved in ethanol, and the solvents from 20HE, surfactants (Tween 20 and Span 80), and CHOL were removed under reduced pressure using high-vacuum rotary evaporation. The residue was dried under vacuum to form a thin dry film, which was subsequently rehydrated with either water or 0.1 M citrate buffer at pH 5.5. To achieve the desired particle size, the niosomes were sonicated with a homogenizer (Homogenizer Sonopuls HD 4100 with an ultrasonic head TS 106, BANDELIN electronic GmbH & Co. KG, Berlin, Germany). A schematic overview of the niosome preparation procedure is shown in Figure 5.

Eight niosomal formulations were developed, composed of Tween 20, Span 80, CHOL, and, where applicable, 20HE, as summarized in Table 1. The A-series samples consisted of Tween 20:Span 80:CHOL mixtures only, with molar ratios of 1:7:2 for 1A, 1:5:1 for 2A, 0.75:6.25:2 for 3A, and 0.5:6:1.5 for 4A, corresponding respectively to concentrations of 1227.5, 3000.2, and 773.3 µg/mL; 1227.5, 2143.0, and 386.7 µg/mL; 920.7, 2678.8, and 773.3 µg/mL; and 613.8, 2571.6, and 580.0 µg/mL for Tween 20, Span 80, and CHOL. The corresponding C-series samples additionally contained 20HE, yielding molar ratios of 1:7:2:1 for 1C, 1:5:1:1 for 2C, 0.75:6.25:2:1 for 3C, and 0.5:6:1.5:1 for 4C. In these formulations, the concentrations of Tween 20, Span 80, and CHOL remained identical to those of the corresponding A-series samples. At the same time, 20HE was incorporated at a constant concentration of 480.6 µg/mL in all C-series formulations. Each formulation in both the A and C series was hydrated with either sterile distilled water, Aqua B. Braun, or 0.1 M citrate buffer at pH 5.5.

Table 1 summarizes the composition of all prepared niosomal formulations, expressed as the molar ratio of Tween 20, Span 80, CHOL, and 20HE.

### 2.3. Niosomes Physicochemical Characterization, Encapsulation Efficiency, Stability, and Impact on Viability of HEK and PHEK Cells

#### 2.3.1. Physicochemical Characterization

Niosome size was measured by nanoparticle tracking analysis (NTA) using a NanoSight LM10 equipped with an sCMOS camera and a 405 nm laser (NTA 3.2 Dev Build 3.2.16, Malvern Panalytical, Malvern, UK). This method determines particle size distribution and concentration by tracking Brownian motion according to the Stokes–Einstein equation. Before analysis, samples were diluted 1:10,000 to match the instrument’s operating range [27].

Particle size and polydispersity index (PDI) were determined using dynamic light scattering (DLS), while zeta potential was measured through electrophoretic light scattering (ELS) with a Zetasizer Ultra (Malvern Panalytical). All measurements were performed in triplicate. To achieve the optimal particle concentration, 10 µL of the niosome suspension was diluted with 10 mL of water [28]. The measurements were conducted in a polymer cuvette, which is recommended for size and PDI analysis. At the same time, the zeta potential was measured using a U-shaped cuvette equipped with a gold electrode.

The pH of the prepared niosomes was measured with a SevenCompact pH meter (Mettler Toledo, Columbus, OH, USA). Before measurement, the instrument was calibrated using standard buffer solutions at pH 4.00 ± 0.05, 7.00 ± 0.00, and 10.00 ± 0.05.

#### 2.3.2. Encapsulation Efficiency

The EE of the active ingredient in the nanoformulation was determined by High-Performance Liquid Chromatography (HPLC) with UV detection. Content and stability analyses were performed on an Agilent 1260 Infinity II LC System (Agilent Technologies, Böblingen, Germany) equipped with a quaternary pump and degasser, a vial sampler, and a diode-array detector (DAD). To determine the amount of compound encapsulated in the nanocarrier, the sample was purified using commercially available size-exclusion chromatography columns (PD Minitrap G25, Cytiva, Marlborough, MA, USA) according to the manufacturer’s instructions. Briefly, the columns were conditioned, and a spin protocol was used to obtain a purified formulation free of unencapsulated active ingredient. Next, a purified sample was mixed with methanol, injected onto a chromatographic column (RP C18) and analyzed using a gradient elution. Phase A consisted of a 0.1% (*v*/*v*) solution of phosphoric acid in water, and phase B consisted of acetonitrile for chromatography. The initial volume-phase ratio was 88:12 (A:B) and changed to 20:80 in 7 min at a flow rate of 0.75 mL/min. For the next 5 min, the gradient elution returned to the initial concentration and maintained the ratio for the next 5 min in isocratic elution. The total run time was 17 min. The 20HE exhibits strong UV absorption with a maximum of approximately 245 nm. All solvents were chromatographic grade and were supplied by Avantor Performance Materials Poland S.A. (Gliwice, Poland).

#### 2.3.3. Stability

The storage stability of the prepared niosomal formulations was assessed over 21 days under refrigerated conditions (2–4 °C). Selected physicochemical parameters, including particle size, PDI, zeta potential, and pH, were measured immediately after preparation (D0) and at 7-day intervals, namely on days 7, 14, and 21 (D7, D14, and D21, respectively). All stability results are presented as mean ± SD. The stability data was interpreted with the support of statistical analysis. Statistical analysis was performed to evaluate time-dependent changes in EE, particle size, PDI, zeta potential, and pH during storage. The values recorded at D7, D14, and D21 days of storage were then compared with the corresponding initial values measured at D0 for each formulation. The analysis was performed using a paired Student’s *t*-test followed by Holm–Bonferroni correction for multiple comparisons. Differences were considered statistically significant when the adjusted *p*-value was <0.05; significance was indicated as * *p* < 0.05 and ** *p* < 0.01.

#### 2.3.4. Viability Assessment—MTS Assay

HEK and PHEK cells were maintained in KGM-Gold™ Keratinocyte Growth Medium BulletKit™ (Lonza, Walkersville, MD, USA) supplemented with 10% FBS.

Cells were cultured under standard conditions (37 °C, 5% CO_2_, 95% humidity). The CellTiter 96^®^ AQueous One Solution Cell Proliferation Assay (MTS; Promega, Madison, WI, USA) was used according to the manufacturer’s recommendations to assess the effects of studied nanoformulations on cell viability. Briefly, the cells were seeded in 96-well plates (5000 cells/well). After 24 h, the growth medium was replaced with fresh medium containing the tested formulations at concentrations ranging from 5 to 75 µM, and the cells were incubated for additional 24, 48, and 72 h. Then, cells were washed with PBS, and 120 µL of freshly prepared MTS solution in complete medium (20 µL of MTS solution + 100 µL of medium) was directly added to each well. After 1-h incubation, the absorbance signal (490 nm) was measured using Tecan SPARK (Multi-mode Microplate Reader, Tecan Trading AG, Männedorf, Switzerland). The assay was performed in triplicate, with three replicates per assay.

### 2.4. Assessment of the In Vivo Toxicity of the 20HE Nanoformulation

According to current law, including Directive 2010/63/EU, studies on *Danio rerio* larvae up to 120 hpf (hours post-fertilization) do not require approval from the Animal Ethics Committee, as they are not considered protected life stages. Fish embryotoxicity was assessed in accordance with the OECD Test Guideline 236 (Fish Embryo Acute Toxicity, FET) [29], with modifications to accommodate behavioral activity assessment. Fertilized embryos of *Danio rerio* were exposed from 0 h post fertilization (hpf) to 96 hpf. Observations were made 24, 48, 72, and 96 hpf. Mortality and sublethal effects were recorded, including lack of embryo development, lack of heart rhythm, lysis, lack of hatching, yolk sac swelling, pericardial edema, morphological deformities, and lack of spontaneous movements. Functional analysis was performed at 96 hpf using the Noldus system (Noldus Information Technology, Wageningen, The Netherlands), recording larval activity for 30 min. The analyzed parameters included distance traveled [mm], speed [mm/s], and mobility [s].

Each study group consisted of 10 individuals subjected to the same conditions. The following study groups were used:

1. control conditions (treated with standard solution E3)
2. positive control—treated with dichloroaniline (toxic)
3. blank nanoformulation 1A/water
4. 20HE 1 mM in nanoformulation 1C/water in final concentration: 1 µM, 2 µM, 5 µM, 10 µM, and 20 µM.

Prior to the encapsulation effect, the effect of 20HE dissolved in DMSO was studied. The following concentrations were tested: 1 μM 20HE/0.1% DMSO, 5 μM 20HE/0.5% DMSO, 10 μM 20HE/1% DMSO, 50 μM 20HE/5% DMSO, 100 μM 20HE/10% DMSO. The DMSO concentrations were determined by the limited solubility of 20HE in this solvent. The need of using DMSO as solubilizer is the effect of 20HE poor water solubility. In the presence of 5% and 10% DMSO, expected damage and mortality of *Danio rerio* embryos were observed [30,31]. In development-safe concentrations of DMSO in the range of 0.01 to 1%, no effect of the presence of 20HE in concentrations of 1 μM, 5 μM, and 10 μM was observed (Supplementary Figure S1).

## 3. Results and Discussion

### 3.1. Assessment of 20HE Biological Activity Based on Cytokine Levels

In this study, the anti-inflammatory activity of free 20HE was evaluated in an in vitro model using PHEK and HEK cell lines. In HEK cells, IL-2, IL-4, IL-5, IL-17A, and IL-22 were either below the assay detection limits, consistent with the low basal inflammatory status of healthy keratinocytes. In contrast, in PHEK cells, all analyzed cytokines were detectable; however, statistically significant modulation by 20HE was observed only for IL-4, IL-17A, and IL-22, whereas IL-2 and IL-5 remained unchanged following treatment. Under these conditions, 20HE did not significantly affect the analyzed cytokines, suggesting that it does not disturb cellular homeostasis in non-inflamed cells. In contrast, detectable levels of IL-4, IL-17A, and IL-22 were observed in PHEK cells, and treatment with 20HE reduced these levels by approximately 20–34%, indicating an anti-inflammatory effect under elevated inflammatory conditions. These findings suggest that 20HE may attenuate inflammatory signaling associated with psoriasis-related cytokine activity, particularly within the IL-17A-associated inflammatory axis.

Hydrocortisone, used as a positive anti-inflammatory control, reduced the level of IL-17A by approximately 30% in PHEK cells. These findings confirmed the responsiveness of the inflammatory model and supported the specificity of the observed effects of 20HE.

**Figure 6:**
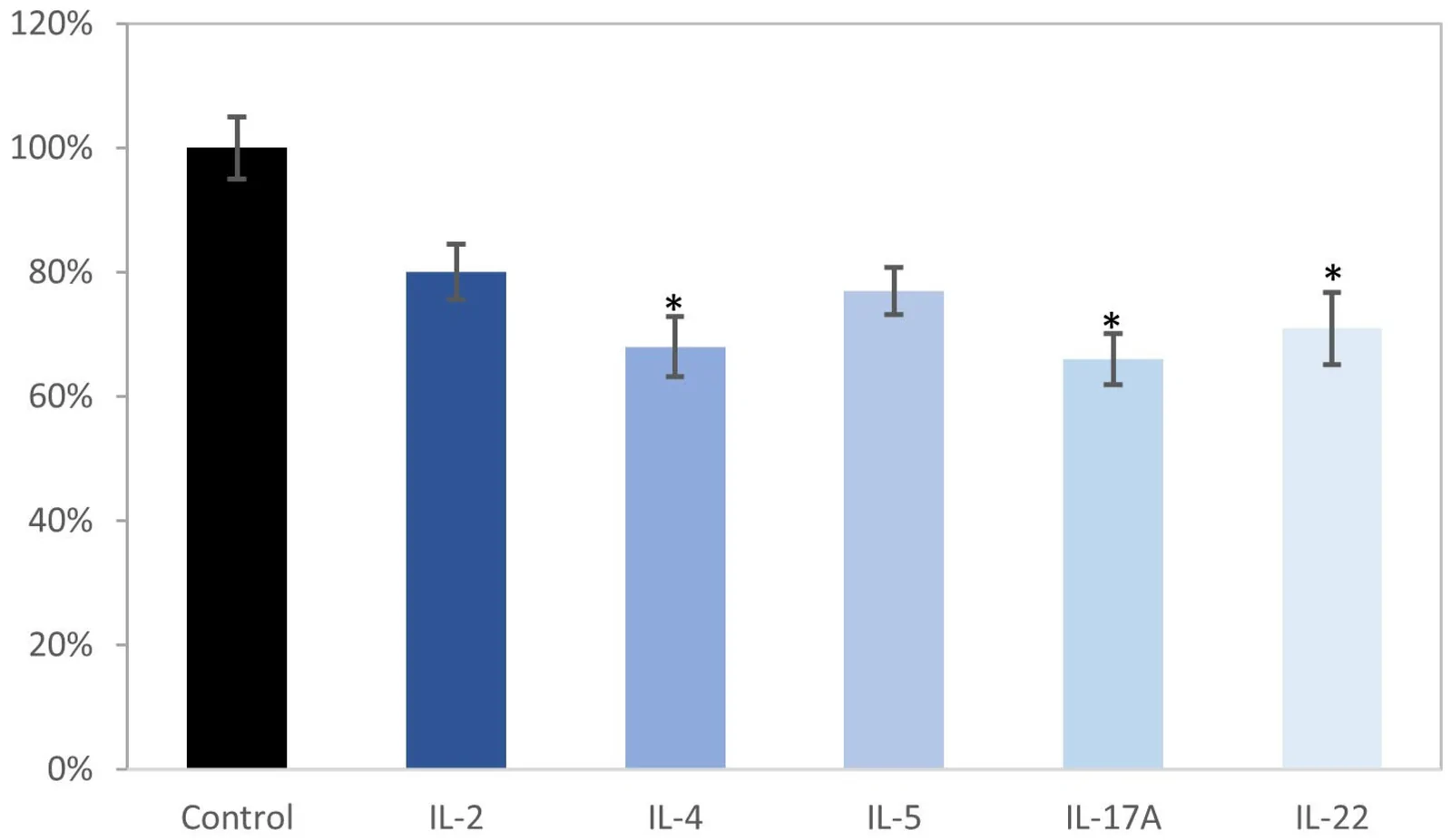
Levels of selected cytokines in PHEK cells following treatment with 20HE. Results are presented as mean ± SEM (*n* = 3). For hydrocortisone (positive control)-treated PHEK cells, the IL-17A level was 70% relative to the untreated control group, for which the level was set to 100%. An asterisk (*) denotes a significant difference compared with untreated cells (Dunnett test, *p* < 0.05).

Figure 6 shows the changes in selected cytokine levels in PHEK cells following treatment with 20HE, expressed as a percentage of the untreated control.

Sun et al. reported that treatment with 20HE, especially at a dose of 20 mg/kg, effectively attenuated the inflammatory cascade and oxidative stress in collagen-induced arthritis rats, suggesting its potential anti-rheumatoid arthritis activity. Treatment with 20HE suppressed activation of the NF-κB signaling pathway and downregulated inflammation-related mediators, including inflammatory markers such as NO, COX-2, and CRP; pro-inflammatory cytokines such as IL-1β, IL-6, and TNF-α; and the inflammatory enzymes iNOS and COX-2 [32]. In another study, momordin Ic, 20HE, and oleanolic acid were shown to significantly suppress PGE_2_ production in LPS-stimulated cells [33].

One limitation of the present study is that the cytokine assay did not include a reference anti-inflammatory compound such as dexamethasone. Since the primary objective was to establish whether 20HE exhibits intrinsic anti-inflammatory activity in psoriatic keratinocytes, untreated cells were used as the experimental reference. Future studies should include clinically established anti-inflammatory agents to enable direct comparisons of efficacy.

### 3.2. Physicochemical Characterization, Encapsulation Efficiency, and Stability of Niosomes

**Figure 7:**
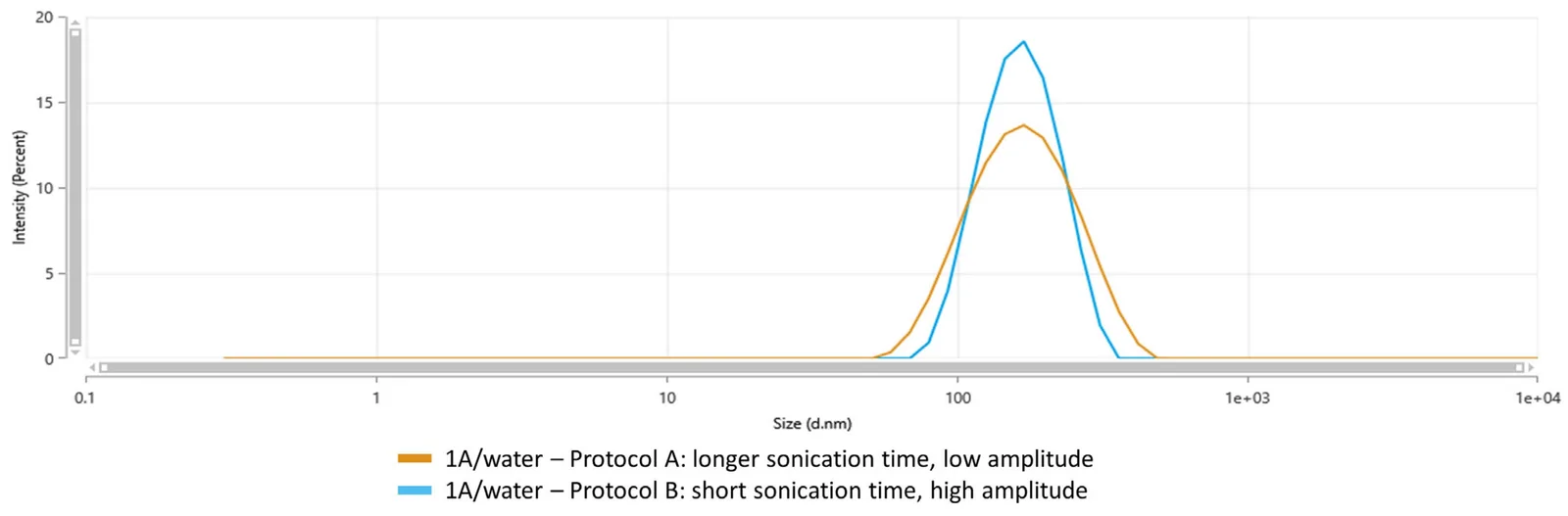
Comparison of DLS intensity-weighted particle size distribution profiles of sample 1A/water obtained after probe sonication using two different protocols: longer sonication time, low amplitude (Protocol A), and short sonication time, high amplitude (Protocol B).

To compare and select the most suitable sonication conditions, water-hydrated niosomes were processed using two different probe sonication protocols: Protocol A, a longer low-amplitude treatment delivering 3.210 kJ, and Protocol B, a shorter high-amplitude treatment delivering 0.2 kJ. Although probe sonication has been widely used as a post-processing approach to reduce niosome size, reported conditions range from 30 s to several minutes, depending on formulation composition and processing parameters [34,35]. In the present study, the substantially shorter Protocol B was sufficient to produce relatively homogeneous vesicles below 300 nm, indicating its potential as a rapid size-reduction strategy. Figure 7 compares the intensity-weighted particle size distributions obtained for the same water-hydrated niosome sample after processing under the two conditions.

Both sonication protocols A and B produced water-hydrated niosomes within the desired size range, with mean particle diameters below 200 nm in both cases (153.2 nm, PDI = 0.214 for Protocol A and 164.5 nm, PDI = 0.264 for Protocol B). Although Protocol A yielded a slightly smaller mean particle size and lower polydispersity, Protocol B was selected for further studies because it also produced niosomes of appropriate size and acceptable homogeneity after production while markedly reducing processing time.

#### 3.2.1. Physicochemical Characterization

The physicochemical characteristics of the prepared niosomal formulations, including particle size, PDI, zeta potential, and pH, were evaluated immediately after preparation (D0). NTA and DLS determined particle size and PDI, whereas ELS measured zeta potential. The results are summarized in Table 2. In general, particle sizes obtained by DLS were higher than those determined by NTA across all formulations, likely due to methodological differences between the techniques. As reported by Filipe et al., NTA complements DLS in particle-size characterization and can be particularly useful for analyzing both monodisperse and polydisperse nanosystems; however, methodological differences between the techniques may lead to discrepancies in the measured size distribution, especially in samples containing particles of different sizes [36]. The differences between NTA- and DLS-derived particle size were more evident in buffer-based formulations than in water-hydrated samples. The average DLS–NTA size difference was approximately 20 nm for water formulations, whereas it increased to approximately 57 nm for buffer formulations, reaching nearly 90–98 nm for 3C/buffer, 4A/buffer, and 4C/buffer. This trend was accompanied by generally higher DLS-PDI values in buffer-based samples.

**Figure 8:**
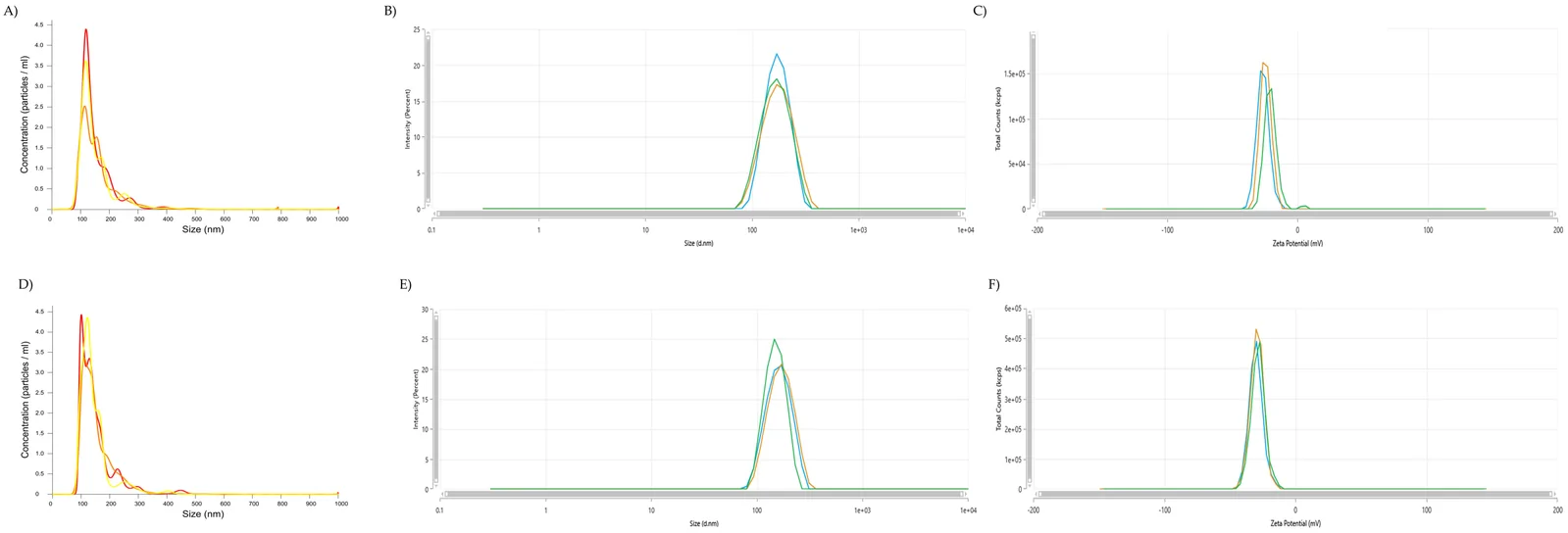
Physicochemical characterization of the prepared niosomal formulations 1A and 1C by NTA (**A,D**), DLS (**B,E**), and ELS (**C,F**). Panels (**A**–**C**) refer to formulation 1A/water and panels (**D**–**F**) to formulation 1C/water, showing NTA particle size distribution profiles, DLS particle size distribution profiles, and ELS zeta potential profiles, respectively.

To provide a more comprehensive characterization of the selected formulations, particle size distribution profiles of formulations 1A/water and 1C/water were analyzed by NTA, DLS, and ELS. Figure 8 presents representative NTA size distributions, DLS particle size distributions, and ELS zeta potential measurements for the selected formulations.

The size of lipid-based vesicles plays a crucial role in the efficiency of skin delivery. Vesicles ≥ 600 nm generally remain in the stratum corneum and, after drying, form a superficial lipid layer, limiting penetration. In contrast, smaller vesicles enhance dermal delivery: particles ≤ 300 nm can reach deeper layers to some extent, while those ≤ 70 nm achieve the highest deposition in both viable epidermis and dermis [37].

Among the water-hydrated formulations, particle sizes determined by NTA ranged from 140.5 to 152.5 nm, indicating a relatively narrow size distribution across the tested systems. The corresponding DLS values were higher, ranging from 147.6 to 194.5 nm. For the buffer-hydrated formulations, NTA-derived particle sizes ranged from 150.7 to 177.7 nm, whereas DLS measurements showed a broader, clearly higher range of 168.0 to 267.0 nm.

Thus, all formulations remained below 300 nm at D0, indicating their potential suitability for topical skin delivery, including psoriasis-related applications. The buffer-hydrated formulations were generally larger than the corresponding water-hydrated systems, particularly when assessed by DLS. This suggests that the hydration medium affected not only vesicle formation, but also the hydrodynamic properties of the particles. The larger particle sizes observed by DLS compared with NTA, particularly for buffer-hydrated formulations, may be attributed to increased vesicle population heterogeneity and the higher sensitivity of intensity-weighted DLS to a small fraction of larger or associated vesicles [36]. The relatively high DLS PDI values of these formulations further support the presence of a broader and more heterogeneous particle-size distribution.

Sguizzato et al. demonstrated that the hydration medium can significantly affect niosome size, lamellarity, and stability. In their study, niosomes prepared with Tween 20 or Span 20 showed medium-dependent differences in vesicle properties: Tween 20 was associated with more hydrated and larger vesicles, particularly when combined with poloxamer 188, whereas Span 20, especially in borate buffer, yielded more stable systems [38]. In our study, the largest NTA–DLS size discrepancies were observed in buffer-hydrated formulations with lower Tween 20 content and predominance of Span 80, especially 3A/buffer, 3C/buffer, 4A/buffer, and 4C/buffer. This indicates that the niosome response to an acidic citrate buffer may be modulated by the Tween 20/Span 80 ratio.

For water-hydrated formulations, NTA-derived PDI values ranged from 0.14 to 0.19, whereas DLS-derived PDI values ranged from 0.24 to 0.35. For buffer-hydrated formulations, PDI values ranged from 0.14 to 0.23 by NTA and from 0.30 to 0.40 by DLS. Therefore, most formulations exhibited acceptable or near-acceptable size distributions. The higher PDI values obtained by DLS, particularly for buffer-hydrated samples, further support the observation that citrate buffer influenced the hydrodynamic properties and apparent heterogeneity of the vesicles. For nanoliposomal formulations, PDI values below 0.5 are generally associated with monodisperse systems, whereas values below 0.3 are considered acceptable and indicative of a homogeneous population of phospholipid vesicles [39]. However, although the latest FDA “Guidance for Industry” for liposome drug products [40] highlights particle size and size distribution as critical quality attributes (CQAs) and key parameters in stability studies, it does not define specific acceptance criteria for PDI values.

A clear medium-dependent effect was observed: niosomes hydrated with citrate buffer were generally larger and exhibited slightly higher polydispersity than those hydrated with water, with this difference being more pronounced in DLS than in NTA measurements. This suggests that the hydration medium affected not only vesicle organization itself, but also the hydrodynamic properties of the particles. The strongest effect was observed for formulations 3 and 4, indicating that the impact of the hydration medium was composition-dependent.

Zeta potential is a key physicochemical parameter that reflects the surface charge and stability of nanoformulation systems. High absolute values of zeta potential (≤−30 mV or ≥+30 mV) indicate strong repulsion between particles, which contributes to good stability and prevents aggregation [26]. All formulations showed negative zeta potential values, ranging from −51.8 to −27.1 mV in water-hydrated formulations, and from −64.7 to −24.3 mV in buffer-hydrated formulations, indicating the predominantly anionic nature of the obtained niosomes. These findings are consistent with previous reports demonstrating that, despite being composed of nonionic surfactants and CHOL, niosomes can still exhibit a negative zeta potential, likely due to ion interaction at the vesicle interface and the negative surface character of CHOL across a wide pH range [34,35]. Most formulations exhibited values around or below −30 mV, suggesting good physical stability, although a few samples showed slightly lower absolute values. The differences between water- and buffer-hydrated niosomes also indicate that the hydration medium influenced surface-related properties of the dispersion, as reflected by changes in zeta potential. This effect was likely further modulated by surfactant composition and ion adsorption at the vesicle interface. Hasani et al. showed that zeta potential depended on surfactant type, with the lowest value observed for Tween 20 niosomes (3.53 mV) and the highest for Span 20 vesicles (17.00 mV). Span 60 also showed a relatively high value (12.2 mV), whereas Tween 80 remained low (3.87 mV), indicating generally higher zeta potential for Span-based than Tween-based niosomes [41]. Tefas et al. also indicated that zeta potential may be influenced by the properties of the hydration medium (phosphate buffer). In their study, liposomes prepared at 45 °C in a medium at pH 4.5 showed higher zeta potentials, whereas those prepared at 55 °C in a medium at pH 5 showed lower surface charge. The authors related this to differences in ionic strength, as the media had molarities of 0.05 M and 0.02 M, respectively, which may have affected charge shielding around the particles [42]. Similarly, in another study, the absolute zeta potential increased with ionic strength, and the highest values were obtained for formulations prepared in PBS at pH 5.6, characterized by an ionic strength of 0.40 M [39].

The pH of dermal formulations is an important parameter influencing skin compatibility. In this study, water and citrate buffer (pH 5.5) were used as hydration media to obtain formulations with pH values close to that of the skin surface. A pH range of 5.5–7.0 can be considered suitable for dermal delivery, as the skin surface is slightly acidic, with a pH of about 5.5, gradually increasing toward neutral values in the deeper skin layers [43]. Maintaining pH within this range helps minimize irritation and preserve the skin barrier. The pH of the water-hydrated samples ranged from 6.27 to 6.82, whereas the buffer-hydrated samples ranged from 5.47 to 5.52. As these values are close to the physiological skin pH, the developed formulations can be considered suitable for topical application. In this respect, it is also worth noting that Manosroi et al. found that niosomal gel and cream (pH in the range of 6.0–7.0) with semi-purified rice bran extracts were non-irritating to rabbit skin over 72 h and improved skin lightening, hydration, thickness, roughness, and elasticity in 30 human volunteers after 28 days [44].

To further contextualize the physicochemical parameters obtained, Table 3 summarizes selected topical and dermal niosomal or niosome-based systems reported in the literature. The comparison includes the preparation method of niosomes, main formulation components, vesicle size, PDI, and zeta potential.

Compared with the literature data, the formulations developed in the present study showed particle sizes within a range commonly reported for topical vesicular systems, remaining below 300 nm in both NTA and DLS measurements. Their PDI values were also within or near the range typically considered acceptable for lipid-based vesicular dispersions, although some formulations showed greater variability. Moreover, the negative zeta potential values observed in the present study were comparable to those reported for several previously studied topical niosomal formulations. Table 4 summarizes key physicochemical parameters and their interpretation in the context of the present study.

#### 3.2.2. Encapsulation Efficiency

Drug concentration was determined using the direct method, in which the drug content is measured in the formulation after its purification from the medium. Following purification by size exclusion chromatography, the isolated formulation was dissolved in methanol and analyzed by HPLC. EE was calculated as the percentage ratio of the drug content measured in the purified sample (by HPLC) to the theoretical drug concentration assumed at the production stage. The HPLC analysis provided valuable insights into the EE and the concentration of the active ingredient (20HE) in the niosomal formulations.

Table 5 presents the concentration (µg/mL) of 20HE. The data show consistent encapsulation across all formulations. Some water-based formulations (1C/water, 3C/water) show slightly higher concentrations compared to their buffer-based counterparts (1C/buffer, 3C/buffer), but the trend is not uniform across all formulations. This suggests that the type of solvent may influence the EE, though differences are generally small.

Concentrations in a purified sample of 20HE were generally comparable between water-hydrated and buffer-hydrated formulations, ranging from 106.8 to 150.6 µg/mL and from 96.2 to 196.0 µg/mL, respectively. Notably, the buffer-hydrated systems exhibited a wider concentration range.

The achieved EE for 20HE was considered satisfactory given the compound’s physicochemical properties. 20HE is a polyhydroxylated ecdysteroid derived from the cholestane skeleton [10,13], which imparts relatively high polarity and limits its affinity for the hydrophobic portion of the lipid membrane. Consequently, its incorporation into niosomes occurs primarily within the limited volume of the aqueous core of the vesicles. The literature on lipid carriers indicates that achieving very high EE for small, hydrophilic molecules is difficult due to the limited volume of the aqueous phase and the potential for diffusion of the substance into the external environment during carrier preparation and purification. Therefore, the obtained EE value is consistent with expectations based on the chemical structure of 20HE [57,58,59].

#### 3.2.3. Stability

The stability of the optimized niosomal formulation was evaluated over 3 weeks at 2–4 °C by monitoring EE, particle size, PDI, zeta potential, and pH.

**Figure 9:**
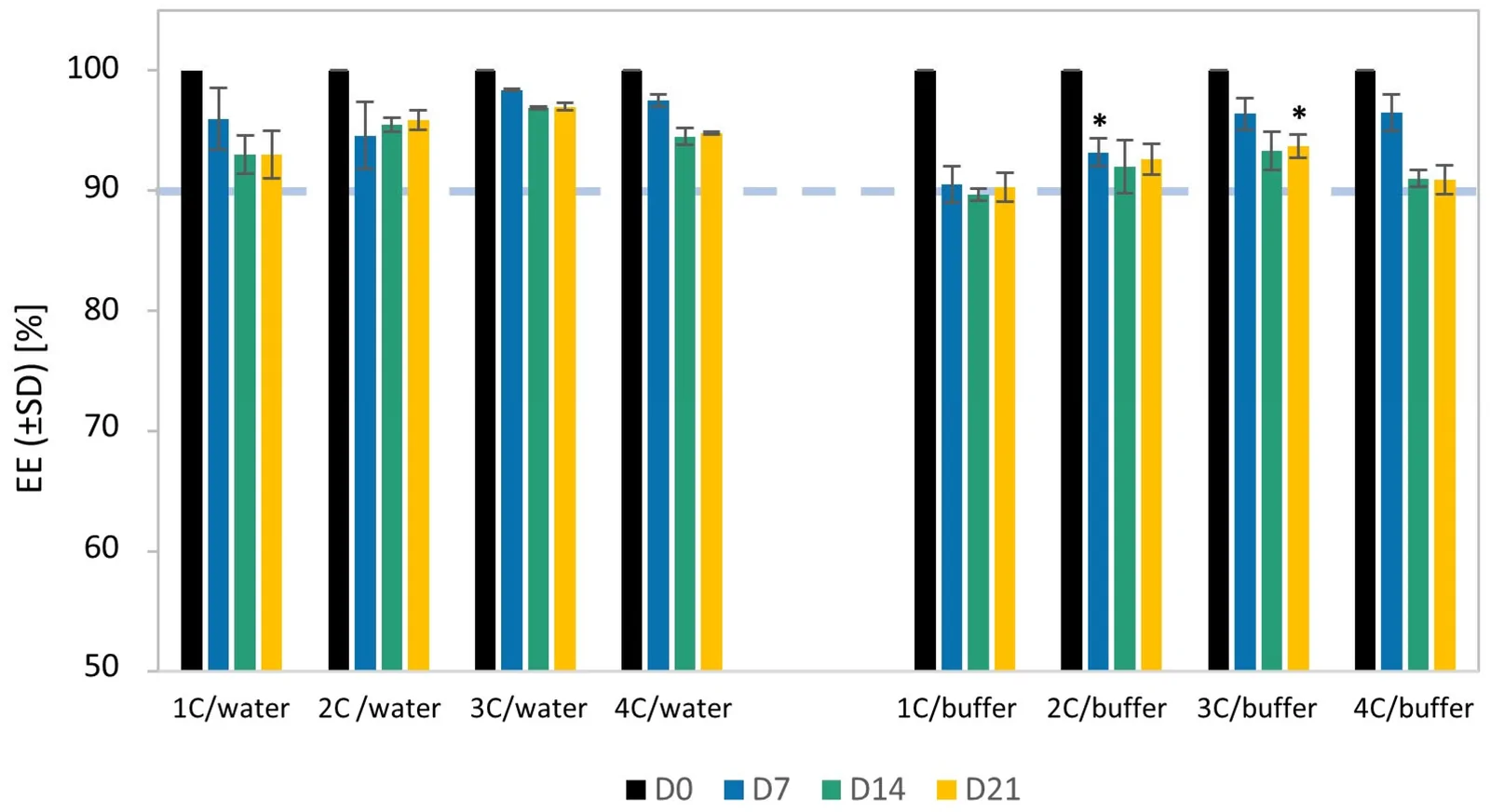
Comparative EE profiles of 20HE-loaded niosomal formulations hydrated with water or buffer during 21 days of storage at 2–4 °C. EE was evaluated for each formulation at D0, D7, D14, and D21. The blue dashed line represents the reference threshold adopted for EE evaluation, corresponding to 90% of the initial EE value measured at D0. Asterisks indicate significant differences compared with D0 after Holm–Bonferroni-corrected paired Student’s *t*-test: * *p* < 0.05.

Figure 9 shows the EE at days D0, D7, D14, and D21. Water-based formulations were considered more stable during the observed time period, as they consistently maintained higher EE values along with lower variability than buffer-based formulations.

Statistical analysis of EE confirmed that most formulations maintained EE values that were not significantly different from their corresponding D0 values after correction for multiple comparisons. After Holm–Bonferroni correction, significant decreases relative to D0 were observed only for the buffer-hydrated formulations 2C/buffer at D7 (adjusted *p* = 0.042, * *p* < 0.05) and 3C/buffer at D21 (adjusted *p* = 0.030, * *p* < 0.05). Nevertheless, EE values remained close to or above the predefined 90% threshold, indicating overall retention of the encapsulated compound during storage.

**Figure 10:**
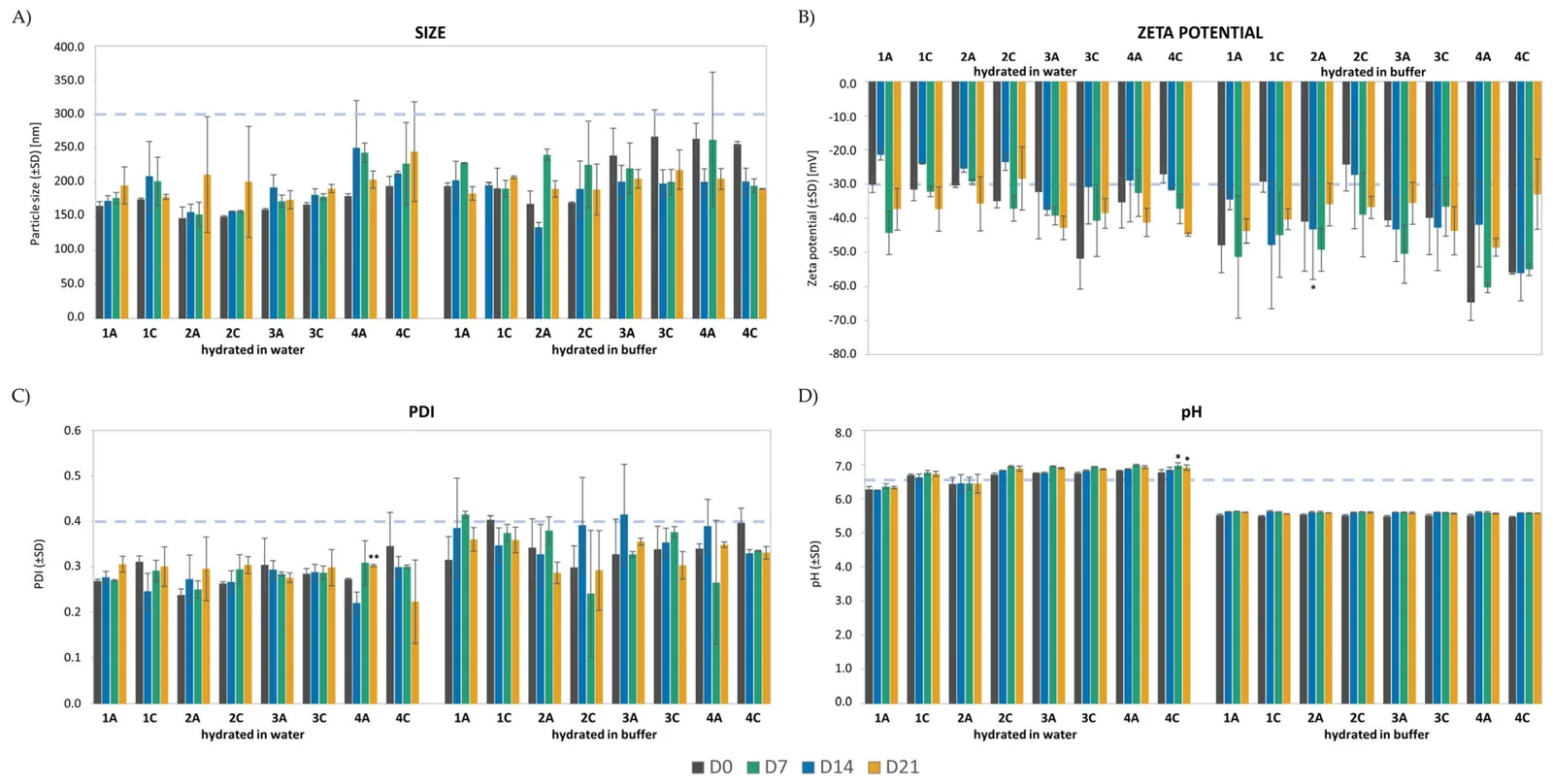
Comparative stability profiles of niosomal formulations hydrated with water or buffer during 21 days of storage at 2–4 °C, based on particle size, PDI, zeta potential, and pH measurements. (**A**) Particle size, (**B**) zeta potential, (**C**) PDI, and (**D**) pH of the formulations at D0, D7, D14, and D21. The blue dashed line indicates the reference threshold used for interpretation of each parameter: 300 nm for particle size, −30 mV for zeta potential, 0.4 for PDI, and pH 6.5 for pH. Asterisks indicate significant differences compared with D0 after Holm–Bonferroni-corrected paired Student’s *t*-test: * *p* < 0.05, ** *p* < 0.01.

Figure 10 presents the stability profiles of the developed niosomal formulations over 21 days of storage at 2–4 °C, evaluated at four time points (D0, D7, D14, and D21) for particle size, PDI, zeta potential, and pH.

Overall, the water-hydrated formulations showed lower particle sizes and lower PDI values than the corresponding buffer-hydrated systems, particularly when assessed by DLS, indicating a more favorable initial physicochemical profile at D0 in terms of vesicle size distribution and homogeneity. In contrast, buffer-hydrated formulations exhibited pH values closer to the physiological pH range of the skin surface. During storage at 2–4 °C, the water-hydrated niosomes generally maintained more consistent physicochemical profiles, with smaller fluctuations in particle size, PDI, and zeta potential observed across several formulations. These findings suggest that both hydration media yielded systems with physicochemical properties suitable for further evaluation as topical nanocarriers, with water hydration conferring greater stability during the study period.

To support the interpretation of the stability data, particle size, PDI, zeta potential, and pH values measured at D7, D14, and D21 were compared with the corresponding D0 values for each formulation using a paired Student’s *t*-test followed by Holm–Bonferroni correction. After correction, most of the analyzed physicochemical parameters did not differ significantly from their initial D0 values.

For water-hydrated formulations, a significant increase in PDI was observed for 4A/water after 21 days of storage (adjusted *p* = 0.004, ** *p* < 0.01). In addition, the pH of 4C/water increased significantly after 14 and 21 days of storage, with adjusted *p*-values of 0.039 and 0.015, respectively (* *p* < 0.05). For buffer-hydrated formulations, a significant change in zeta potential was observed for 2A/buffer after 7 days of storage (adjusted *p* = 0.032, * *p* < 0.05).

Water-hydrated formulations showed smaller fluctuations in particle size, PDI, and zeta potential during storage. In contrast, buffer-hydrated formulations exhibited more frequent decreases in EE and greater variability in selected physicochemical parameters.

Phosphate buffer is the most frequently employed hydration medium in niosomal formulations. Flurbiprofen-loaded niosomes showed the highest entrapment efficiency at pH 5.5 [60]. The selection of acidic citrate buffer was supported by the findings of Burgalassi et al., who reported that a pH 5.5 citrate buffer solution enhanced the EE of oleuropein/HP-β-cyclodextrin-loaded liposomes compared with a pH 7.4 phosphate buffer solution. EE increased in both ultrasonicated and extruded citrate-buffer-hydrated formulations compared with PBS-hydrated ones, from 69.63 ± 1.02% to 72.98 ± 5.62% and from 77.64 ± 3.08% to 80.77 ± 1.35%, respectively. Moreover, citrate-buffer hydration resulted in favorable vesicle-size characteristics, including a main population of 242.4 ± 38.19 nm after ultrasonication and, after extrusion, a smaller size and lower PDI than PBS hydration: 235.5 ± 14.94 nm and 0.310 ± 0.0317 versus 268.2 ± 6.53 nm and 0.362 ± 0.0268, respectively [61]. Although this observation was obtained for liposomal systems, it suggested that an acidic citrate environment may be beneficial for vesicle-based formulations. In our preliminary experiments, PBS-hydrated 20HE-loaded niosomes exhibited poor physical stability and loss of dispersion homogeneity. Therefore, an acidic citrate buffer was selected over PBS as the buffered hydration medium and subsequently compared with water during the optimization of 20HE-loaded niosomes. This strategy was investigated for its potential to improve vesicle stability and its limited use in niosomal formulations. Similar stability during storage at 4 °C was also reported by Junyaprasert et al. for ellagic acid niosomes composed of surfactants, CHOL, and stabilizing and solubilizing agents. The formulations, which additionally contained either dimethyl sulfoxide (DMSO) or *N*-methyl-2-pyrrolidone (NMP) as penetration enhancers, remained stable for 4 months at 4 °C. Moreover, DMSO-based niosomes were found to be more effective for epidermal delivery of ellagic acid, whereas NMP-based niosomes were more suitable for dermal delivery [50]. Interestingly, Isnan and Jufri developed green tea extract-loaded niosomes to improve antioxidant stability and skin permeation. Formulations with surfactant-to-CHOL molar ratios of 3:1 (F1), 2:1, 1:1 (F3), and 0.5:1 were prepared. Although F1 showed the highest encapsulation, it underwent phase separation after 7 days. After incorporation into hydroxypropyl methylcellulose as the gelling agent, the F3 formulation exhibited the best physical stability, with no noticeable changes [48].

### 3.3. Viability Assessment—MTS Assay

**Figure 11:**
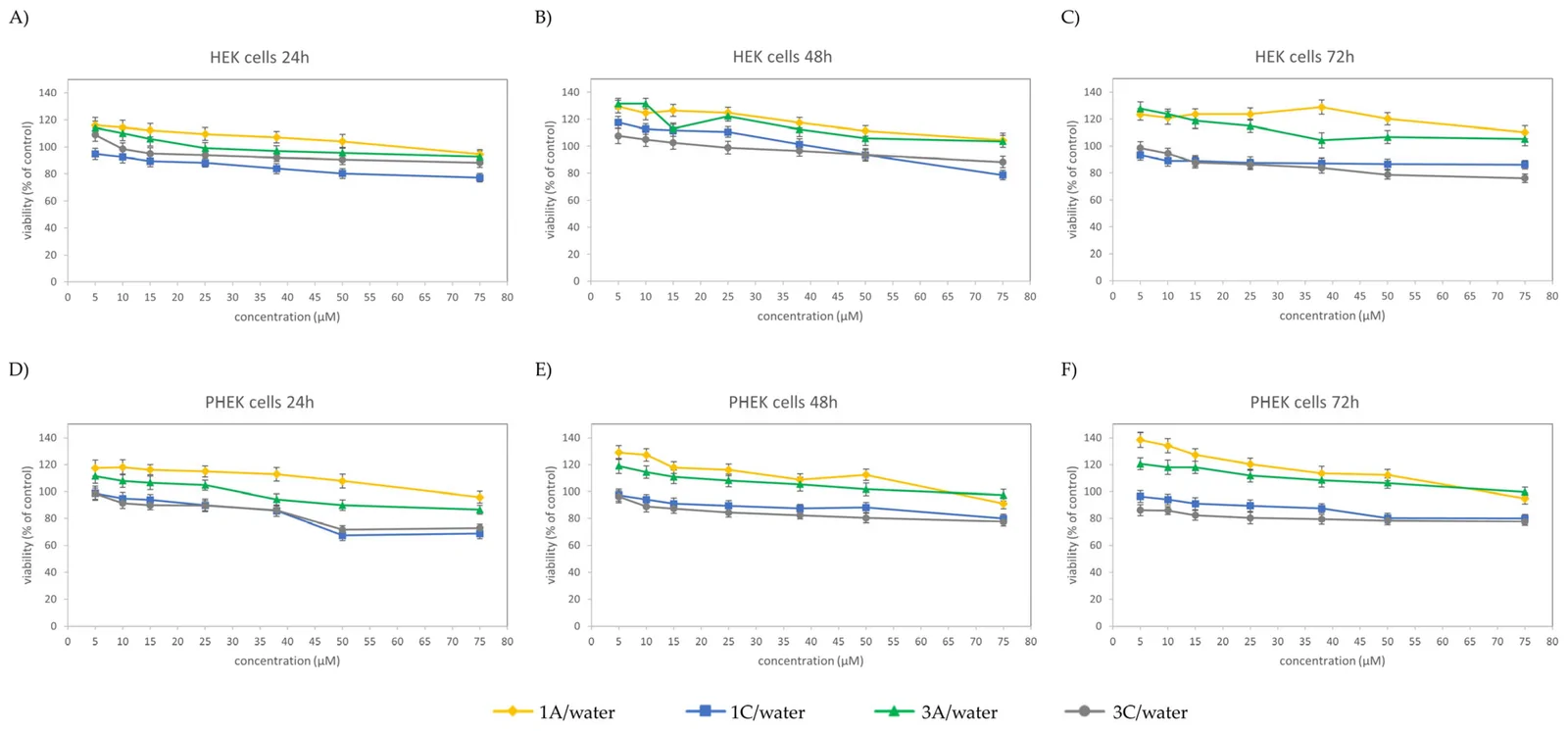
Viability of HEK and PHEK cells after incubation with water-hydrated 20HE formulations measured by MTS. HEK cells were exposed to 1A/water, 1C/water, 3A/water, and 3C/water for 24 h (panel (**A**)), 48 h (panel (**B**)), and 72 h (panel (**C**)), while PHEK cells were exposed for 24 h (panel (**D**)), 48 h (panel (**E**)), and 72 h (panel (**F**)). The tested formulations were applied at concentrations ranging from 5 to 75 µM. Data are expressed as the mean and SEM based on three biologically independent replicates.

Cell viability was assessed in HEK and PHEK cells following exposure to 1A/water, 1C/water, 3A/water, and 3C/water at concentrations ranging from 5 to 75 µM for 24, 48, and 72 h. The MTS assay demonstrated concentration- and time-dependent effects of the tested compounds in both cell lines. The effect of the tested formulations on HEK and PHEK cell viability is presented in Figure 11.

In HEK cells, nanoformulation containing 20HE exhibited limited cytotoxicity after 24 h, while longer exposure resulted in increased metabolic activity at lower concentrations and decreased viability at higher doses. A similar trend was observed in PHEK cells, although these cells appeared to be more sensitive to selected treatments. In particular, nanoformulation containing 20HE induced mild, concentration-dependent reductions in cell viability, with more pronounced effects in PHEK cells compared with HEK cells.

Importantly, none of the tested compounds reduced cell viability below 50% at any concentration or time point in either cell line, and all calculated IC_50_ values exceeded 75 µM under the experimental conditions applied (Table 6).

20HE is considered a promising ingredient for dry and very dry skin, especially in conditions associated with impaired keratinocyte differentiation [12]. Thus, MTS assay results are important for assessing its cellular compatibility in topical formulations.

The literature also confirms that niosomal carriers can effectively enhance skin permeation and improve topical therapeutic performance. In niosomal systems, the choice of surfactant and edge activator is an important determinant of vesicle deformability, stability, and drug permeation. Compounds such as Span 80, Span 20, Tween 20, and sodium cholate have been reported to modulate vesicle flexibility, thereby affecting transdermal or cellular drug delivery [62]. Kheilnezhad and Hadjizadeh further suggested that surfactants with longer hydrophobic chains are better suited for the incorporation of hydrophobic drugs and for promoting intracellular delivery, whereas surfactants with larger hydrophilic head groups may facilitate the penetration of hydrophilic compounds [63]. In our formulation, Span 80 was used in a higher proportion than Tween 20. This composition is justified by the long hydrophobic alkyl chain of Span 80, which favors the incorporation of lipophilic compounds, while Tween 20, owing to its bulky hydrophilic head group, may contribute to vesicle stabilization and interaction with the aqueous phase.

According to Priprem et al., loading the anthocyanin complex into niosomes significantly extended its anti-inflammatory activity in a dose-dependent manner, with a pronounced effect observed 8 h after topical use, supporting the suitability of niosomes for topical anti-inflammatory applications [64].

Manosroi et al. showed that papain-loaded niosomes, particularly elastic niosomes, improved transdermal delivery of the drug and enhanced scar healing. After 28 days, the elastic niosomal papain gel produced a markedly greater reduction in hypertrophic scars on rabbit ears than the gel base, free papain gel, and non-elastic niosomal papain gel, with improvements of 10.20-, 2.73-, and 2.31-fold, respectively. Histological analysis showed that treatment with elastic niosomal papain gel significantly reduced both collagen fiber density and scar height compared with the control group [47].

Shah et al. reported that a gamma-oryzanol niosomal gel showed four times greater permeation and better retention in the dermal layers than the conventional gel [65]. Ex vivo studies using Franz diffusion cells on newborn pig skin demonstrated that ethanol-injection-derived niosomes enhanced resveratrol penetration into the epidermis and dermis more effectively [49]. Kumar and Rai demonstrated that an optimized curcumin-loaded proniosomal gel is non-toxic and non-irritant. However, its anti-inflammatory and anti-arthritic activities were lower than those of marketed indomethacin products [66].

### 3.4. Assessment of the In Vivo Activity of the 20HE Nanoformulation

The *Danio rerio* model has become an important platform in dermatological research because its genetic relatedness to humans, together with its distinctive physiological features, makes it well-suited for investigating skin diseases. *Danio rerio* serves as a practical intermediate model between in vitro cell studies and in vivo investigations. Owing to, among others, its low maintenance requirements, high fecundity, transparent embryos, short generation time, rapid embryonic development, and relevance to human disease phenotypes [67,68,69]. Tokarz et al. described *Danio rerio* as a widely recognized model organism in developmental biology, while noting that the influence of steroid hormones on embryonic development has not yet been fully elucidated [70].

**Figure 12:**
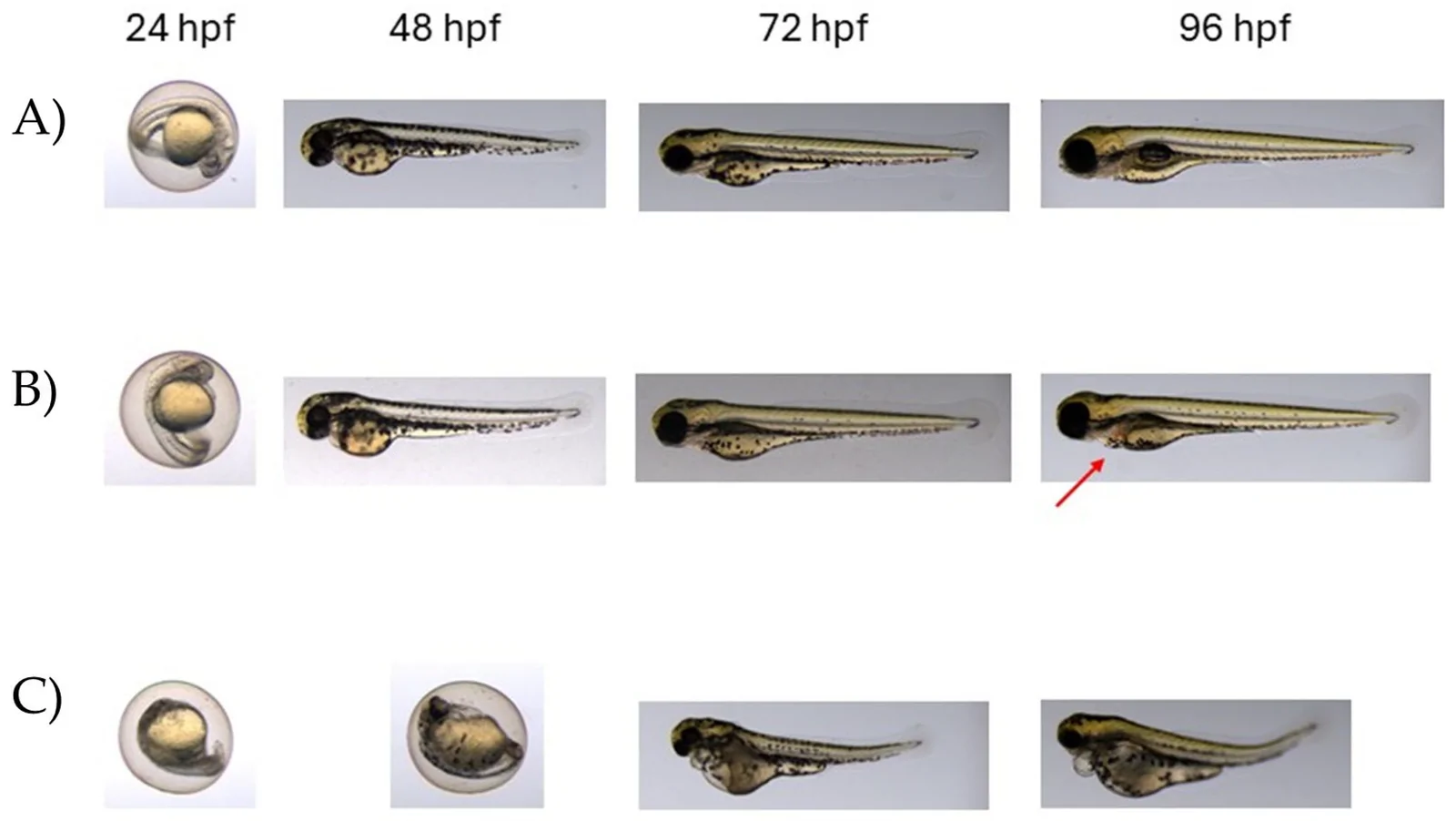
Embryos of *Danio rerio* development during exposure from 0 h post fertilization (hpf) to 96 hpf in the control condition (**A**), with 20 µM 20HE in nanoformulation (**B**), and dichloroaniline (**C**). Observations were made 24, 48, 72, and 96 hpf. The red arrow indicates pericardial edema.

The 1A/water carrier induced sublethal effects following prolonged exposure (96 hpf), including developmental abnormalities of the eyes, tail, and swim bladder, suggesting time-dependent toxicity potentially associated with the non-ionic surfactants Tween 20 and Span 80. However, as the individual excipients were not evaluated separately, their specific contributions to the observed effects cannot be conclusively determined. The frequency of developmental changes in this group ranged from ~10% to ~60%. At increasing concentrations of the test substance, i.e., 20HE, a slight severity of these disorders was observed only at 96 hpf. Shorter exposure to 20HE did not affect embryo development. The only effect that can be attributed to the action of 20HE is the swelling of the pericardium that occurs after 96 hpf in 10–20% of embryos. Behavioral parameters remained comparable to those of controls across the full range of 20HE concentrations. The Lowest Observed Effect Concentration (LOEC) for 20HE was 20 µM. Figure 12. presents representative images of *Danio rerio* embryos at different developmental stages under control conditions and after exposure to 20 μM 20HE nanoformulation or dichloroaniline.

Considering that 3,4-dichloroaniline is a well-established positive control in *Danio rerio* embryo toxicity testing and its toxicity at early developmental stages is well documented, the limited effects observed in our study suggest that 20HE exhibits relatively low developmental toxicity [71].

The available literature on 20HE in fish models appears very limited. Yang et al. identified β-ecdysterone as one of the constituents potentially responsible for the anti-osteoporotic activity of raw and salt-processed *Achyranthes bidentata*. In the *Danio rerio* model, β-ecdysterone showed marked activity, significantly increasing the mineralized area at 1 μM and also improving cumulative optical density [72]. Interestingly, Cocci et al. reported preliminary findings suggesting that 20HE may influence metabolic status in teleost fish, possibly via FXR as a target and through the interplay between FXR and PPARα [73].

## 4. Conclusions

Assessment of 20HE’s biological activity based on cytokine levels demonstrated that 20HE did not alter cytokine expression in non-inflamed HEK cells, indicating preservation of cellular homeostasis under physiological conditions. In contrast, 20HE reduced the levels of IL-4, IL-17A, and IL-22 in PHEK cells, confirming its anti-inflammatory activity under elevated inflammatory conditions.

The niosomal formulations developed in this work showed physicochemical characteristics favorable for dermal administration. At D0, the particle sizes of all formulations remained below 300 nm, which is considered beneficial for skin delivery. Their dispersity was acceptable, as indicated by the obtained PDI values, and all systems showed predominantly negative zeta potentials, supporting their physical stability. In addition, the pH values of both water-hydrated and citrate buffer-hydrated systems were within or close to the physiological range of the skin surface, indicating suitability for topical use. A comparison of the formulations showed that water-hydrated niosomes had a more advantageous initial physicochemical profile and better storage stability than the corresponding buffer-hydrated systems. HPLC analysis, combined with size-exclusion sample preparation, confirmed the incorporation of 20HE into all tested formulations. Additionally, the niosomal formulations were stable during 21 days of storage at 2–4 °C.

The in vivo study on the *Danio rerio* embryo model showed that the 1A/water caused sublethal developmental abnormalities at 96 hpf, whereas 1C/water (with 20HE) caused only mild pericardial edema after prolonged exposure. No significant behavioral changes were observed, and the LOEC for 20HE was established at 20 μM.

The findings of this preliminary study indicate that 20HE modulates pro-inflammatory cytokine levels, supporting its further evaluation as a potential candidate for topical psoriasis therapy. The developed niosomal formulations exhibited physicochemical characteristics potentially favorable for topical administration. However, their skin delivery performance and in vitro drug-release characteristics have not yet been established, and this constitutes a limitation of the present study. Further studies, including skin permeation, skin deposition, therapeutic efficacy, and extended stability assessments, are therefore required to confirm their suitability for topical application, particularly following incorporation into a final dosage form such as a hydrogel.

## Figures and Tables

**Table 1:** Composition of the prepared niosomal formulations, expressed as molar ratios of Tween 20, Span 80, CHOL, and 20HE. Formulations labeled A correspond to control samples without 20HE, whereas formulations labeled C contain 20HE. The suffix “water” denotes hydration with sterile distilled water, Aqua B. Braun, while “buffer” denotes hydration with 0.1 M citrate buffer at pH 5.5.

Formulation	Molar Ratio			
	Tween 20	Span 80	CHOL	20HE
1A/water and 1A/buffer	1	7	2	-
1C/water and 1C/buffer	1	7	2	1
2A/water and 2A/buffer	1	5	1	-
2C/water and 2C/buffer	1	5	1	1
3A/water and 3A/buffer	0.75	6.25	2	-
3C/water and 3C/buffer	0.75	6.25	2	1
4A/water and 4A/buffer	0.5	6	1.5	-
4C/water and 4C/buffer	0.5	6	1.5	1

**Table 2:** Physicochemical characteristics of the prepared niosomal formulations were determined by NTA, DLS/ELS, and pH meter including particle size, PDI, zeta potential, and pH. Formulations hydrated with water or buffer and marked with A represent control niosomes, while formulations marked with C contain 20HE. Data are presented as mean ± SD.

Sample	NTA		DLS + ELS			pH ± SD
	Particle Size (±SD) [nm]	PDI ± SD	Particle Size (±SD) [nm]	PDI ± SD	Zeta Potential (±SD) [mV]	
1A/water	149.3 ± 64.9	0.19 ± 0.04	165.5 ± 6.2	0.27 ± 0.00	−30.1 ± 2.4	6.3 ± 0.1
1C/water	151.0 ± 65.8	0.19 ± 0.04	175.2 ± 1.2	0.31 ± 0.01	−31.6 ± 3.3	6.7 ± 0.0
2A/water	144.2 ± 62.1	0.19 ± 0.06	147.6 ± 16.1	0.24 ± 0.01	−30.4 ± 0.6	6.4 ± 0.2
2C/water	145.3 ± 60.2	0.17 ± 0.04	149.8 ± 1.5	0.26 ± 0.00	−35.0 ± 2.0	6.7 ± 0.0
3A/water	145.0 ± 58.7	0.16 ± 0.04	159.8 ± 1.3	0.30 ± 0.06	−32.3 ± 13.7	6.7 ± 0.0
3C/water	152.5 ± 57.5	0.14 ± 0.05	166.9 ± 3.3	0.28 ± 0.01	−51.8 ± 8.9	6.7 ± 0.0
4A/water	150.1 ± 61.9	0.17 ± 0.03	179.7 ± 3.4	0.27 ± 0.00	−35.4 ± 7.4	6.8 ± 0.0
4C/water	140.5 ± 59.3	0.18 ± 0.00	194.5 ± 14.4	0.35 ± 0.07	−27.1 ± 2.5	6.8 ± 0.1
1A/buffer	166.1 ± 63.7	0.15 ± 0.01	194.5 ± 4.5	0.32 ± 0.05	−47.9 ± 8.1	5.5 ± 0.0
1C/buffer	164.1 ±67.9	0.18 ± 0.08	195.8 ± 3.7	0.40 ± 0.01	−29.4 ± 3.0	5.5 ± 0.0
2A/buffer	150.7 ± 63.9	0.18 ± 0.01	168.0 ± 19.5	0.34 ± 0.06	−41.0 ± 14.6	5.5 ± 0.0
2C/buffer	151.9 ± 73.0	0.23 ± 0.03	170.0 ± 0.8	0.30 ± 0.05	−24.3 ± 7.7	5.5 ± 0.0
3A/buffer	159.2 ± 61.9	0.15 ± 0.04	239.7 ± 39.7	0.33 ± 0.08	−40.6 ± 1.8	5.5 ± 0.0
3C/buffer	177.7 ± 78.1	0.19 ± 0.01	267.0 ± 39.1	0.34 ± 0.05	−39.9 ± 10.8	5.5 ± 0.0
4A/buffer	168.9 ± 64.0	0.14 ± 0.04	263.5 ± 23.4	0.34 ± 0.01	−64.7 ± 5.3	5.5 ± 0.0
4C/buffer	158.1 ± 63.5	0.16 ± 0.04	255.8 ± 3.9	0.40 ± 0.03	−56.0 ± 0.4	5.5 ± 0.0

**Table 3:** Literature comparison of selected topical and dermal niosomal formulations with respect to preparation method, composition, particle size, PDI, and zeta potential. Where mean ± SD values were reported in the original articles, only mean values are shown for clarity. “—” indicates that the parameter was not reported or was not available in the cited source.

Author	Active Compound	Aim	Method	Main Components	Vesicle Size (nm)	PDI	Zeta Potential (mV)
Manosroi et al. [44]	Rice bran bioactive compounds	To develop topical anti-aging formulations with niosome-entrapped rice bran bioactive compounds	Supercritical carbon dioxide fluid method	Tween 61, CHOL, ferulic acid, γ-oryzanol, and phytic acid	480.9	1.5	—
Junyaprasert et al. [45]	Ellagic acid	To develop ellagic acid-loaded niosomes for dermal delivery	Reverse-phase evaporation	Span 60/Tween 60 surfactant mixtures, CHOL, PEG 400, propylene glycol, or methanol	124–752	<0.4	—
Marzio et al. [46]	Ammonium glycyrrhizinate	To prepare deformable surfactant vesicles for dermal delivery of ammonium glycyrrhizinate	Thin-film hydration and high-speed stirring (dry lipid film hydrated with HEPES buffer solution, pH 7.4, mixed with ethanol)	Tween 20, dicetyl phosphate, CHOL	363–622	0.2–0.3	−23.7 to −3.28
Manosroi et al. [47]	Papain	To develop papain-loaded niosomal gels for enhanced transdermal delivery and scar treatment	Thin-film hydration (dry lipid film hydrated with papain solution in phosphate buffer, pH 7.0)	Tween 61, CHOL, and sodium cholate as an edge activator for elastic niosomes	Non-elastic: 441.8; elastic: 520.2	Non-elastic: 0.515; elastic: 0.593	Non-elastic: −46.2; elastic: −44.5
Isnan and Jufri [48]	Green tea extract	To develop green tea extract-loaded niosomes for improved antioxidant stability and skin permeation	Thin-film hydration (dry lipid film hydrated with phosphate buffer, pH 5.5) and gel incorporation	Span 60, CHOL	~338.3	0.349	−40
Pando et al. [49]	Resveratrol	To formulate resveratrol-loaded niosomes for topical delivery	Thin-film hydration (dry lipid film hydrated with deionized water) or the ethanol injection method	Gelot 64, oleic acid, and linoleic acid	TFH-S: 293–496; EIM: 284–402	TFH-S: 0.30–0.40; EIM: 0.28–0.40	—
Junyaprasert et al. [50]	Ellagic acid	To develop ellagic acid-loaded niosomes for dermal delivery and evaluate the effect of penetration enhancers on their physicochemical properties	Reverse-phase evaporation	Span 60, Tween 60, CHOL, Solulan C24, PEG 400, and DMSO or NMP	312–402	<0.4	—
Dadashzadeh et al. [51]	*Aloe vera*	To develop a sustained-release niosomal hydrogel for skin regeneration and wound-dressing applications	Reverse-phase evaporation and hydrogel incorporation	Span 60, CHOL	270.080	0.108	—
Ghazwani et al. [52]	Carvacrol oil	To develop a carvacrol oil-loaded niosomal gel for topical anti-inflammatory delivery	Thin-film hydration (dry lipid film hydrated with PBS, pH 6.8)	Tween 80, CHOL	180.23	0.265	−31.70
Tabatabaie-Mehr et al. [43]	Adapalene	To develop adapalene-loaded niosomal hydrogel for topical acne treatment	Thin-film hydration (dry lipid film hydrated with phosphate buffer, pH 7.4)	Span 60, cetyltrimethylammonium bromide, CHOL	168.8	0.273	−70.6
Thongphachanh et al. [53]	Purple waxy corn cob extract	To develop purple-waxy-corn-cob-extract-loaded niosomes for UVB protection and anti-melanogenesis activity	Thin-film method (dry lipid film hydrated with phosphate buffer, pH 5.5, containing 10% propylene glycol)	Span 20, CHOL, propylene glycol	185–296	0.14–0.42	−32 to −29
Saeedi et al. [54]	Menadione sodium bisulfite	To develop a menadione-loaded niosomal formulation for skin-lightening applications	Ultrasonication method	Tween 20, Span 20, CHOL	298.133–653.766	0.3047–0.536	−8.156 to −3.056
Eren Boncu et al. [55]	Baicalein	To develop baicalein-loaded niosomes and niosomal gel for topical antioxidant delivery	Thin-film hydration	Tween 60 or Tween 80, CHOL	591.4–1037.0	0.084–0.505	−44.2 to −12.6
Alkilani et al. [56]	Ascorbic acid and caffeine	To develop niosome-loaded microneedles for dermal co-delivery of antioxidant compounds	Thin-film hydration and microneedle incorporation	Span 60, Tween 60, and Tween 80, CHOL, DCP	130.6–411.2	0.005–0.256	−50.20 to −25.40

**Table 4:** Summary of the key physicochemical parameters and their relationship to the results obtained in the present study.

Parameter	Relevance to Skin Delivery	Preferred Value/Implication	Present Study (D0) for Water-Hydrated Niosomes	Present Study (D0) for Buffer-Hydrated Niosomes
Size	Determines skin penetration depth	↓ size = ↑ skin deposition; ≤300 nm favorable for dermal delivery; ≤70 nm associated with the highest deposition; ≥600 nm mainly retained on the skin surface	140.45–152.50 nm (NTA), 147.62–194.52 nm (DLS)	150.65–177.70 nm (NTA), 167.98–267.00 nm (DLS).
PDI	Indicates vesicle population homogeneity	↓ PDI = ↑ homogeneity; ≤0.3 (≤0.4) generally acceptable for a homogeneous formulation	0.143–0.191 (NTA), 0.238–0.346 (DLS)	0.144–0.231 (NTA), 0.299–0.404 (DLS)
Zeta potential	Reflects stability and aggregation tendency	↑ |ZP| = ↑ stability; values ≤ −30 mV or ≥+30 mV suggest good physical stability	−51.84 to −27.14 mV	−64.67 to −24.30 mV
pH	Influences skin compatibility and tolerability	pH close to the physiological skin range (4.5–6.5) is preferred for topical application	6.27–6.82	5.47–5.52

**Table 5:** Sample concentrations (µg/mL) of the active ingredient (20HE) for each formulation (formulation type: water-based or buffer-based).

Sample	Concentration (µg/mL) ± SD
1C/water	150.6 ± 1.1
2C/water	121.0 ± 5.0
3C/water	123.0 ± 1.0
4C/water	106.8 ± 0.2
1C/buffer	126.8 ± 1.2
2C/buffer	196.0 ± 4.7
3C/buffer	96.2 ± 0.8
4C/buffer	121.7 ± 0.5

**Table 6:** IC_50_ values of the tested formulations in HEK and PHEK cells.

Formulation	HEK IC_50_	PHEK IC_50_
1A/water	>75 µM	>75 µM
1C/water	>75 µM	>75 µM
3A/water	>75 µM	>75 µM
3C/water	>75 µM	>75 µM

## Data Availability

The data are contained within the article.

## Supplementary Materials

The following supporting information can be downloaded at https://www.mdpi.com/article/10.3390/pharmaceutics18091096/s1. Figure S1. Embryos of *Danio rerio* development during exposure from 0 hours post fertilization (hpf) to 96 hpf with 1 μM 20HE in 0.1% DMSO in E3, 5 μM 20HE in 0.5% DMSO in E3, 10 μM 20HE in 1% DMSO in E3. Observations were made at 24, 48, 72, and 96 hpf.

## Abbreviations

The following abbreviations are used in this manuscript:

20HE	20-hydroxyecdysone
DDS	drug delivery systems
DLS	dynamic light scattering
DMSO	dimethyl sulfoxide
DOX	doxorubicin
EE	encapsulation efficiency
ELS	electrophoretic light scattering
FBS	fetal bovine serum
GPCR	G protein-coupled receptor
HEK	human epidermal keratinocytes/normal human epidermal keratinocytes
hpf	hours post fertilization
HPLC	High-Performance Liquid Chromatography
IL-2	interleukin 2
IL-4	interleukin 4
IL-5	interleukin 5
IL-17A	interleukin 17A
KGM	Keratinocyte Growth Medium
LOEC	Lowest Observed Effect Concentration
MTT	3-(4,5-dimethylthiazol-2-yl)-2,5-diphenyltetrazolium bromide
NMP	N-methyl-2-pyrrolidone
NTA	nanoparticle tracking analysis
PDI	polydispersity index
PHEK	psoriasis patient-derived keratinocyte
PI3K	phosphoinositide 3-kinase
PLC	phospholipase C
Th17	T helper 17
