# Supplementary material for: 20-Hydroxyecdysone-Loaded Niosomes as a Novel Nano-Therapeutic Platform for Psoriasis: In Vitro Characterization and Danio rerio Toxicity Studies
Source: Pharmaceutics. 2026 Aug 31;18(9):1096. doi: 10.3390/pharmaceutics18091096 (PMC13610785; doi:10.3390/pharmaceutics18091096)
Supplement: Supplementary file 1 [file pharmaceutics-18-01096-s001.zip › Supplementary-Figure S1.pdf]

Supplementary Information

The following concentrations of 20HE/DMSO were tested:

1. *1 µM 20HE / 0,1% DMSO*
2. *5 µM 20HE / 0,5% DMSO*
3. *10 µM 20 HE / 1% DMSO*
4. *50 µM 20HE / 5% DMSO*
5. *100 µM 20HE / 10% DMSO*

The DMSO concentrations were determined by the limited solubility of 20HE in this solvent. As expected, damage and mortality of Danio rerio embryos were observed in the presence of 5% and 10% DMSO [1,2]

In development-safe concentrations of DMSO in the range of 0.01 to 1 %, no effect of the presence of 20HE in concentrations of 1 µM, 5 µM, and 10 µM was observed (Supplementary Fig. 1). Therefore, selected concentrations were used in the actual experiment.

1. Hoyberghs et al., 2021, DMSO Concentrations up to 1% are Safe to be Used in the Zebrafish Embryo Developmental Toxicity Assay. DOI: 10.3389/ftox.2021.804033;
2. Xiong et al., 2017, Comparative Developmental Toxicity and Stress Protein Responses of Dimethyl Sulfoxide to Rare Minnow and Zebrafish Embryos/Larvae. DOI: 10.1089/zeb.2016.1287.

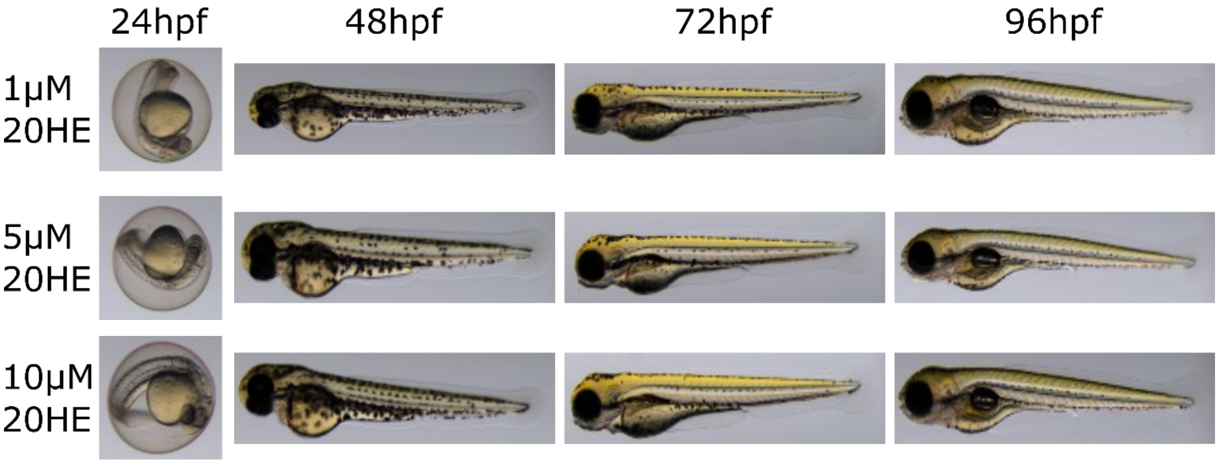

Supplementary Figure S1. Embryos of Danio rerio development during exposure from 0 hours post fertilization (hpf) to 96 hpf with 1 µM 20HE in 0.1% DMSO in E3, 5 µM 20HE in 0.5% DMSO in E3, 10 µM 20HE in 1% DMSO in E3. Observations were made at 24, 48, 72, and 96 hpf.
